# PartIES: a disease subtyping framework with Partition-level Integration using diffusion-Enhanced Similarities from multi-omics Data

**DOI:** 10.1093/bib/bbae609

**Published:** 2024-11-25

**Authors:** Yuqi Miao, Huang Xu, Shuang Wang

**Affiliations:** Department of Biostatistics, Mailman School of Public Health, Columbia University, New York, NY 10027, United States; Department of Biostatistics, Mailman School of Public Health, Columbia University, New York, NY 10027, United States; Department of Biostatistics, Mailman School of Public Health, Columbia University, New York, NY 10027, United States

**Keywords:** multi-omics integration, disease subtyping, similarity-based methods, diffusion, partition-level similarity learning

## Abstract

Integrating multi-omics data helps identify disease subtypes. Many similarity-based methods were developed for disease subtyping using multi-omics data, with many of them focusing on extracting common clustering structures across multiple types of omics data, but not preserving data-type-specific clustering structures. Moreover, clustering performance of similarity-based methods is affected when similarity measures are noisy. Here we proposed PartIES, a Partition-level Integration using diffusion-Enhanced Similarities to perform disease subtyping using multi-omics data. PartIES uses diffusion to reduce noises in individual similarity/kernel matrices from individual omics data types first, and then extract partition information from diffusion-enhanced similarity matrices and integrate the partition-level similarity through a weighted average iteratively. Simulation studies showed that (1) the diffusion step enhances clustering accuracy, and (2) PartIES outperforms competing methods, particularly when omics data types provide different clustering structures. Using mRNA, long noncoding RNAs, microRNAs expression data, DNA methylation data, and somatic mutation data from The Cancer Genome Atlas project, PartIES identified subtypes in bladder urothelial carcinoma, liver hepatocellular carcinoma, and thyroid carcinoma that are most significantly associated with patient survival across all methods. Further investigations suggested that among subtype-associated genes, many of those that are highly interacting with other genes are known important cancer genes. The identified cancer subtypes also have different activity levels for some known cancer-related pathways. The R code can be accessed at https://github.com/yuqimiao/PartIES.git

## Introduction

Disease subtyping using molecular profiles led to numerous discoveries [1–3]. Integrating multiple types of molecular profiles such as genomic, epigenomic, and transcriptomic profiles provides a better understanding of biological mechanisms and a more accurate subtyping [4]. Clustering methods like K-means, partition around medoids, and hierarchical clustering have been widely used. With high-dimensional omics data, dimension reduction methods, such as principal component analysis (PCA), non-negative matrix factorization (NMF) [5] and auto-encoder [6] have been applied to learn low-dimensional feature representations before applying aforementioned clustering methods. With multi-omics data, many early methods for disease subtyping are model-based such as Gaussian latent variable models, e.g. iCluster [7] and its extensions [8, 9], which assume a common cluster structure across data types. More recent methods tend to learn feature representations on multi-omics data first and integrate them followed by K-means. For example, iNMF [10] applies NMF and pattern fusion analysis [11] uses PCA to learn feature representations of individual omics data types first, which are then averaged with weights followed by K-means. Deep learning methods such as auto-encoder were also used for feature representations on individual omics data types, which are then concatenated for K-means clustering [12].

Another category of methods is similarity-based methods [13–18]. For these methods, pairwise similarities between subjects are calculated using features of each omics data type, and multiple similarity matrices/graphs are integrated into an overall similarity matrix, on which spectral clustering is conducted, where eigenvectors of the graph Laplacian induced by a similarity graph are extracted, and K-means is applied on these graph representations. Many similarity-based subtyping methods with multi-omics data construct individual similarity matrices from individual omics data types through kernels and focus on ways to integrate similarity matrices/kernels with the assumption that individual omics data types provide similar clustering structures. For example, similarity network fusion (SNF) [13] iteratively averages elements in one similarity matrix with elements in other similarity matrices as weights until convergence and then can be averaged. Another popular method CIMLR (Cancer Integration via Multi-kernel LeaRning) [16] learns an overall similarity matrix from a weighted average of individual similarity matrices/kernels with a low-rank constraint, where larger weights are learned for kernels with structures closer to the overall similarity, aiming to extract a consensus clustering pattern across data types.

However, disease subtyping studies have suggested that different omics data types might provide distinct cluster structures [7, 19, 20]. A breast cancer subtyping study identified different subtypes with distinct survival patterns using mRNA expression and DNA methylation data separately [19]. An ovarian cancer subtyping study suggested that subtypes identified using gene expression data only partially agree with those found using DNA methylation and microRNA expression data, respectively [20]. These data-type-specific cluster structures might be overlooked by some current similarity-based integration methods or iCluster and its extensions that assume similar clustering structures across data types. Recently, methods were also developed that first extract clustering information from individual omics data types and then integrate them [21–23]. For example, PINS (Perturbation clustering for data INtegration and disease Subtyping) [21] obtains individual pairwise connectivity matrices using clusters identified from individual omics data types and averages them to have an overall connectivity matrix followed by a similarity-based clustering method. More recently, partition-level Multi-View Clustering [22] was developed for imaging data that first extracts partition information from individual similarity matrices and integrates similarities on the partition level. When different omics data types provide distinct clustering structures, we want methods that can preserve data-type-specific cluster structures before integration. Moreover, as clustering results of similarity-based methods are greatly affected when similarity measures are noisy, different network diffusion methods [24–26] were developed to reduce noise or variances of similarity measures using neighbor information.

Here we developed PartIES, a Partition-level Integration framework that uses diffusion-Enhanced Similarities. PartIES first conducts diffusion on individual similarity matrices from individual omics data types to reduce noises and then partitions diffusion-enhanced similarity matrices to capture distinct data-type-specific cluster structures and integrates low-rank partition-information-induced similarity matrices through a weighted average iteratively. We conducted extensive simulation studies to evaluate clustering performance of PartIES and competing methods SNF and CIMLR, with/without diffusion. We applied PartIES and competing methods to identify subtypes of three cancers: bladder urothelial carcinoma (BLCA), liver hepatocellular carcinoma (LIHC), and thyroid carcinoma (THCA) using mRNAs, long noncoding RNAs (lncRNAs), and microRNAs (miRNAs) expression data, DNA methylation data, and somatic mutation data from the The Cancer Genome Atlas (TCGA) project. Subsequent survival analyses suggest that BLCA, LIHC, and THCA subtypes identified by PartIES are most significantly associated with patient survival across all methods. We further investigated biological meanings of the identified subtypes and noticed that among subtype-associated genes, many of those that are highly interacting with other genes are known important cancer genes. The identified cancer subtypes also have different activity levels for some known cancer-related pathways.

## Methods

**Figure 1:**
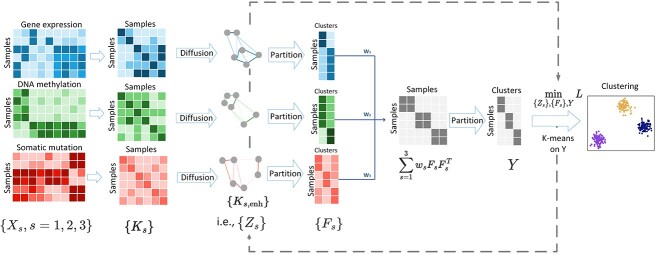
The schematic plot of PartIES: (1) construct individual kernel/similarity matrices from original feature matrices, (2) enhance individual similarity/kernel matrices through diffusion, and (3) extract partition information from individual diffusion-enhanced kernels and integrate individual partition-level similarity matrices with a weighted average iteratively by minimizing the loss function $L$.

Figure 1 displays the schematic flowchart of PartIES: (1) construct individual similarity/kernel matrices from individual feature matrices; (2) diffusion on individual similarity matrices; and (3) learn a partition-level integrative similarity iteratively.

### Diffusion-enhanced similarity matrices

Given a feature matrix $X_{s,n\times p_{s}}$, $s = {1,...,S}$, where $n$ is the number of subjects and $p_{s}$ is the number of features of data type $s$, we calculate the pairwise similarity measure between subjects $i$ and $j$ using the following kernel function [15]:


(1) \begin{align*}& \begin{split} K_{s}(i,j) &= \exp\left\{-\frac{\|x_{s,i}-x_{s,j}\|_{2}^{2}}{2\epsilon_{s}(i,j)^{2}}\right\} \\ \epsilon_{s}(i,j) &= \mu_{s,i}+\mu_{s,j}, \mu_{s,i} = \frac{\sum_{l\in N_{s,k(i)}}\|x_{s,i}-x_{s,l}\|_{2}}{k}, \end{split}\end{align*}

where $x_{s,i}$ is subject $i$’s feature vector of data type $s$, $\|x\|_{2}$ is the L2-norm of a vector $x$, $N_{s,k(i)}$ is the $k$ nearest neighbors of subject $i$ using data type $s$, $\mu _{s,i}$ is the average Euclidean distance between subject $i$ and his(her) $k$ nearest neighbors using data type $s$, and $\epsilon _{s}(i,j)$ is the sum of $\mu _{s,i}$ and $\mu _{s,j}$, which reflects similarity ranges based on neighbors of subjects $i$ and $j$ using data type $s$. We further express kernel distances between subjects $i$ and $j$ as $D_{K_{s}}(i,j) = K_{s}(i,i)+K_{s}(j,j)-2K_{s}(i,j)$.

For data type $s$, to denoise $K_{s}$ using diffusion, we define a local similarity matrix $P_{s}$ using $k$ nearest neighbors: $P_{s}(i,j) = \frac{K_{s}(i,j)\cdot I\{j\in N_{s,k(i)}\}}{\sum _{l \in N_{s,k(i)}}K_{s}(i,l)}$, and obtain the diffusion-enhanced similarity matrix through a one-hop diffusion: $K_{s}P_{s}^{T}$. To have symmetric enhanced similarity matrices, we further define $K_{s,enh} = (K_{s}P_{s}^{T}+P_{s}K_{s})/2$, and the corresponding $D_{K_{s,enh}}$.

### The proposed PartIES

With enhanced kernel distance matrices $D_{K_{s,enh}}, s = 1,\cdots , S$, we propose the following loss function to capture overall cluster structure integrating multiple omics data types while preserving clustering structures from individual data types:


(2) \begin{align*} \min_{\{\{Z_{s}\}, \{F_{s}\}, Y\}} &\sum_{s=1}^{S} \Big\{ < D_{K_{s,enh}}, Z_{s}>_{F}+ \beta||Z_{s}||_{F}^{2} \nonumber\\& + \gamma (tr(F_{s}^{T}(I-Z_{s})F_{s}) + w_{s}||YY^{T}-F_{s}F_{s}^{T}||_{F}^{2}) \Big\} \nonumber\\& s.t. \sum_{j=1}^{n} Z_{s}(i,j)=1, Z_{s}(i,j)\geq 0; F_{s}^{T}F_{s} = I_{C_{s}}; \nonumber\\Y^{T}Y & =I_{C}; i = 1, 2,\cdots, n; s = 1, 2,\cdots, S, \end{align*}

where $Z_{s, n\times n}$ and $F_{s, n\times C_{s}}$ are the similarity matrix and corresponding partition information from data type $s$ with $C_{s}$ the number of clusters using data type $s$, $Y_{n\times C}$ is the integrated partition information with $C$ the number of clusters using all $S$ data types. The choices of $C_{s},s=1,...,S$ and $C$ are guided by the eigengap criterion with details included in the Supplementary Materials. Here $\beta $ and $\gamma $ are non-negative hyper-parameters, which are set such that only $k$ nearest neighbors of sample $i$ have nonzero similarities in $Z_{s}$ during optimization. That is, $Z_{s}$ is a local similarity matrix. Details on tuning $\beta $ and $\gamma $ are included in the Supplementary Materials. Weights $w_{s}$ are calculated as $w_{s} = \frac{1}{2||YY^{T}-F_{s}F_{s}^{T}||_{F}}, s=1,...,S$ with the constraint of summing to 1 and the rationale that the more similar a data-type-specific cluster structure is to the common cluster structure, the more contribution this data type should have in integration.

The first term in the objective function is the Frobenius inner product between the enhanced kernel distance $D_{K_{s,enh}}$ and the learned local similarity matrix $Z_{s}$ for data type $s$, which aims to learn small similarities when distances are large. The second term is a regularization term to avoid learned $Z_{s}$ being identity matrices. If there are $C_{s}$ clusters using data type $s$, molecular profiles of samples in the same cluster should have high similarity, and the effective rank of $Z_{s}$ should be ideally $C_{s}$. The third term along with the constraint on $F_{s}$ enforces the low-rank structure of $Z_{s}$ to preserve the data-type-specific cluster structures [27]. Minimizing the third term is equivalent to performing spectral clustering [28] on $Z_{s}$, which extracts $F_{s, n\times C_{s}}$, the partition information of data type $s$, i.e. the first $C_{s}$ eigenvectors from the graph Laplacian $I-Z_{s}$. We then have $2F_{s}F_{s}^{T}$ as the partition-level similarity matrix for data type $s$. The last term learns the integrated partition information $Y_{n\times C}$ using all $S$ data types, assuming $C$ clusters, where minimizing this term is equivalent to performing spectral clustering on the weighted average $\sum _{s}w_{s}2F_{s}F_{s}^{T}$.

### Optimization

We optimize $Z_{s, n\times n}$ and $F_{s,n\times C_{s}}$ for data type $s, s = 1,\cdots , S$, and $Y_{n\times C}$. Although the objective function is non-convex, optimizing each parameter while holding others constant is convex. We use the following optimization steps:

**Steps 0: initialize $Z_{s}$, $F_{s}$, and $Y$.** With enhanced kernel distance matrix $D_{K_{s, enh}}$, we initialize $Z_{s}(i,j) = -D_{K_{s, enh}}(i,j)+\max _{i,j}(D_{K_{s, enh}}(i,j))$ for $s=1,...,S$. We initialize $F_{s}$ as the first $C_{s}$ eigenvectors of $I-Z_{s}$ corresponding to the $C_{s}$ smallest eigenvalues for $s=1,\cdots ,S$, which is equivalent to extracting partition information from individual diffusion-enhanced kernels using spectral clustering. We initialize $Y$ as the first $C$ eigenvectors of $I-\sum _{s=1}^{S}w_{s}2F_{s}F_{s}^{T}$ with weights being initialized as $w_{s}=\frac{1}{S}$ for $s=1,\cdots , S$.

We then iteratively update each parameter as follows:

**Step 1: update $Z_{s}$ while fixing $F_{s}$, for $s=1,\cdots ,S$ separately:**


\begin{align*}& \begin{split} &\min_{Z_{s}} \{<D_{K_{s,enh}},Z_{s}>_{F}+\beta||Z_{s}||_{F}^{2} +\gamma tr(F_{s}^{T}(I-Z_{s})F_{s}) \} \\ &\text{s.t.} \; \sum_{j} Z_{s}(i,j) = 1, Z_{s}(i,j) \geq 0 \text{ for} i=1,\cdots, n. \nonumber \end{split}\end{align*}

This step localizes $Z_{s}$ with each row having around $k$ non-zero elements with details in the Supplement Materials.

**Step 2: update $F_{s}$ while fixing $Z_{s}$ and $Y$, for $s=1,\cdots ,S$ separately:**


\begin{align*}& \begin{split} &\min_{F_{s}} \left \{tr(F_{s}^{T}(I-Z_{s})F_{s})+ w_{s} ||YY^{T}-F_{s}F_{s}^{T}||_{F}^{2}\right \} \\ & \text{s.t.} \; F_{s}^{T}F_{s} = I_{C_{s}}, \nonumber \end{split}\end{align*}

where $w_{s} = \frac{1}{2\|YY^{T}-F_{s}F_{s}^{T}\|_{F}}$ is calculated using values from the last iteration with normalization $w_{s} = \frac{w_{s}}{\sum _{s=1}^{S} w_{s}}$, and $F_{s, n\times C_{s}}$ is the first $C_{s}$ eigenvectors of $(I-Z_{s}) + w_{s}(I-2YY^{T})$ corresponding to the $C_{s}$ smallest eigenvalues.

**Step 3: update $Y$ while fixing $F_{s}, s = 1,\cdots ,S$:**


\begin{align*}& \begin{split} &\min_{Y} \sum_{s=1}^{S} w_{s}||YY^{T}-F_{s}F_{s}^{T}||_{F}^{2} \\ & \text{s.t.} \; Y^{T}Y = I_{C}, \nonumber \end{split}\end{align*}

where $w_{s} = \frac{1}{2||YY^{T}-F_{s}F_{s}^{T}||_{F}}$ and $Y_{n\times C}$ is the first $C$ eigenvectors of $I-\sum _{s=1}^{S} w_{s}2F_{s}F_{s}^{T}$ corresponding to the $C$ smallest eigenvalues.

Steps are repeated iteratively until converge. The final cluster labels are obtained by performing k-means on $Y_{n\times C}$.

## Simulation studies

We conducted simulation studies to investigate (1) how diffusion on the similarity matrix affects clustering performance with one data type; and (2) the overall clustering performance of PartIES and competing methods SNF and CIMLR, with/without diffusion on individual similarity matrices before integration.

We set each data type to have 10 000 features composed of signal and noise features. We simulated signal features in data type $s$ for subjects in cluster $j$ from a normal distribution $N(\mu _{s,j}, \sigma ^{2}), s=1,...,S, j=1,...,C_{s}$, and noise features from $N(0,1)$ for all subjects. We assumed all signal features from one data type have the same effect size for simplicity. We set the number of neighbors $k=n/4$ and explored different choices of $k$ and included results in the Supplementary Materials. For SNF and CIMLR, we set the number of clusters $C$ as the truth. For PartIES, the choice of $C_{s}, s=1,..., S$ was guided by the eigengap criterion as detailed in the Supplementary Materials, while $C$ was also set as the true number of clusters. We conducted 1000 simulations for each simulation setting and evaluated clustering performance in simulation studies using normalized mutual information (NMI).

### Simulation settings

To investigate if diffusion helps clustering performance, we considered one data type with $n=150$ and three equal-sized clusters with 50 subjects each. We considered two simulation settings varying effect sizes: (1) varying the number of signal features $ \in \{40, 70, 100, \cdots , 220, 250\}$ out of 10,000 features, where all signal features have the same effect size and can separate all three clusters, i.e. signal features were generated from normal $N(1,1)$, $N(2,1)$, and $N(3,1)$ for samples in the three clusters, respectively; (2) fixing the number of signal features as 180 and varying standard deviation (SD) $\sigma \in \{0.1, 0.6, 1.1, 1.6, \cdots , 5.6\}$, where signal features were generated from $N(1, \sigma ^{2})$, $N(2, \sigma ^{2})$ and $N(3, \sigma ^{2})$ for samples in the three clusters, respectively. We conducted spectral clustering on similarity matrices with/without diffusion.

To examine overall clustering performance of PartIES, SNF, and CIMLR, we considered three data types all being generated from normal distributions each with 10,000 features. We set $n=200$ with four equal-sized clusters each with 50 subjects. We set each data type to have the same number of signal features ranging from 10 to 200 with a grid of 10. We considered the following three simulation settings.

In simulation setting I, three data types provide the same clustering structures with signal features in one data type having the same effect size while signal features in different data types have similar effect sizes. Specifically, signal features in data type 1 have means for four clusters $(0.5,0.8,1.5,2)$; signal features in data type 2 have means for four clusters $ (0.5,1.2,1.5,2)$; and signal features in data type 3 have means for four clusters $(0.5,1,1.3,2)$.

In simulation setting II, three data types provide similar clustering structures but signal features in different data types have different effect sizes. Specifically, signal features in data type 1 have means for four clusters $(0.5,1,1.5,2)$, i.e. data type 1 separates all four clusters while it separates clusters 1 and 4 the best. Signal features in data type 2 have means for four clusters $(2,0.5,1,1.5)$, i.e. data type 2 separates clusters 1 and 2 the best. Signal features in data type 3 have means in four clustering $(1.5,2,0.5,1)$, i.e. data type 3 separates clusters 2 and 3 the best. PartIES would perform well in this setting.

In simulation setting III, three data types provide different cluster structures. Specifically, signal features in data type 1 mainly separate clusters 1 and 2 from clusters 3 and 4 with means in four clusters $ (1,1.5,3,3.5)$. Signal features in data type 2 mainly separate clusters 1 and 2 from clusters 3 and 4, and can further separate cluster 1 and cluster 2 with means in four clusters being $(1,2,3,3)$. Signal features in data type 3 separate clusters 1 and 2 from clusters 3 and 4, and can further separate cluster 3 and cluster 4 with means in four clusters being $(3,3,1,2)$. This is the setting PartIES is designed for.

We also conducted simulation studies with the three data types mimicking real data more realistically. Specifically, features in data type 1 were generated from Bernoulli distributions to mimic mutation presence/absence data and features in the other two data types were generated from normal distributions to mimic gene expression data. We considered 200 subjects with four equal-sized clusters each with 50 subjects and 60 subjects from four equal-sized clusters each with 15 subjects and repeated the three simulation settings above. Details of these additional simulation studies were included in the Supplementary Materials.

### Simulation results

**Figure 2:**
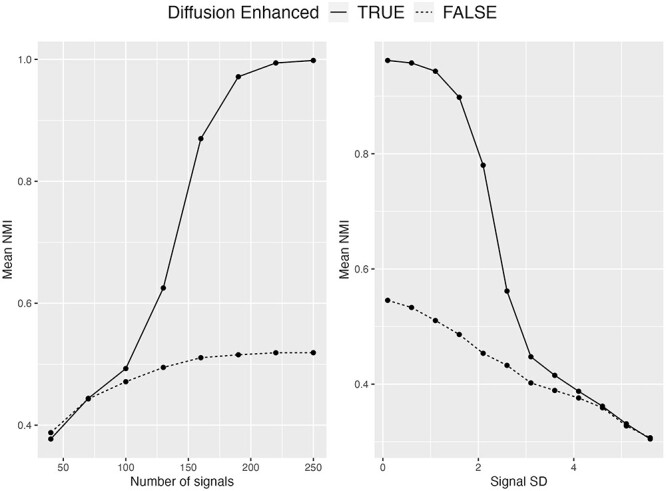
Effect of the proposed diffusion step with mean NMIs across 1000 simulations.

**Clustering performance with diffusion** Figure 2 displays NMI means with/without diffusion for the two simulation settings varying (1) the number of signal features, and (2) $\sigma $ of signal features. We can see that when effect sizes are small, i.e. when the number of signal features is smaller than 70 or $\sigma $ of signal features is greater than 4, clustering performance with/without diffusion are very similar. As effect sizes increase, better clustering performance is observed with diffusion as expected. This is because when effect sizes increase, $k$ nearest neighbors can be more accurately defined. Thus, the diffusion step that relies on neighbors can better help denoise.

**Figure 3:**
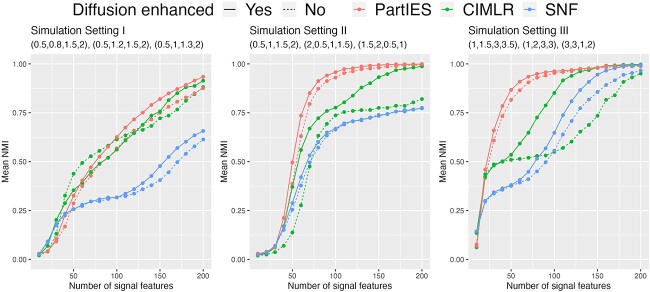
Clustering performance of PartIES and competing methods with mean NMI across 1000 simulations for the three simulation settings considered.

**Clustering performance of PartIES and competing methods** Figure 3 displays NMI means of PartIES and competing methods for the three simulation settings considered. First of all, we can see that the diffusion step improves the clustering accuracy of all methods in the three simulation settings considered, especially in setting II when three data types provide similar clustering structures but signal features in the three data types have different effect sizes, and in setting III when three data types provide different clustering structures. Moreover, the diffusion step helps CIMLR more because the original CIMLR does not use local similarities from individual data types, while the original SNF uses local similarities when fusing multiple similarity matrices from multiple data types. Similarly, PartIES also imposes local similarities in the step of learning $Z_{s}$.

For the overall clustering performance, under simulation setting I when three data types provide the same cluster structures and signal features in different data types have similar effect sizes, PartIES and CIMLR have similar performance and are much better than that of SNF. This is because the granular differences in clustering structures due to small differences in effect sizes in different data types are not captured by the SNF fusing step that uses individual similarity matrices directly. Under setting II when three data types provide similar cluster structures but signal features in different data types have different effect sizes and setting III when three data types provide different cluster structures, PartIES that integrates data-type-specific partition information performs much better than the two competing methods as expected.

For the additional simulation studies with three data types being generated from different distributions and with a much smaller sample size, we observed very similar results as being reported above. Details were included in the Supplementary Materials and Figs S3–4.

## Real data applications

### Data processing

We applied PartIES and competing methods with/without diffusion to identify BLCA, LIHC, and THCA subtypes using mRNA, lncRNA, miRNA expression, DNA methylation and somatic mutation data from TCGA. We downloaded omics data using the R package ‘TCGAbiolinks’ and used tumors with all five omics data types, leading to 401 BLCA tumors, 362 LIHC tumors, and 484 THCA tumors (Table 1). The original 450K DNA methylation data have measures on 485,577 CpGs. We excluded CpGs (1) on sex chromosomes, (2) overlapping with known single nucleotide polymorphisms, and (3) with > 30% missing rates. We ended up with 405 336 CpGs in BLCA data, 399 476 CpGs in LIHC data, and 415 378 CpGs in THCA data. We further corrected type I/II probe bias using the R package ‘wateRmelon’ and imputed missing values using k-nearest neighbors. There is no missingness in three types of gene expression data and somatic mutation data. Note that somatic mutation data summarizes numbers of non-synonymous mutations per gene per tumor sample. We summarized numbers of features of each omics type in Table 1. After data processing steps, we normalized each feature with a mean 0 and an SD 1.

**Table 1:** Summary of TCGA omics data for BLCA, LIHC, and THCA tumors

Cancer Types		BLCA	LIHC	THCA
Number of tumor samples		401	362	484
Number of deaths		175	132	14
Median survival days		1008	1694	NA
Number of omics features after QC	mRNA expression	19 962	19 962	19 962
	lncRNA expression	16 901	16,901	16 901
	miRNA expression	1881	1,881	1881
	DNA methylation	405 336	399 476	415 378
	Somatic mutation	16 299	12 761	3193

### Overall performances of PartIES

Table 2 displays cancer subtypes identified by PartIES and competing methods and corresponding log-rank $P$-values that associate subtypes and patient survival. We can see that subtypes identified by PartIES are most significantly associated with patient survival among all methods for all three cancers BLCA, LIHC and THCA. The six BLCA subtypes by PartIES are associated with patient survival with a $P$-value $2.30\times 10^{-5}$. The four LIHC subtypes by PartIES are associated with patient survival with a $P$-value $9.08\times 10^{-4}$. The four THCA subtypes by PartIES are associated with patient survival with a $P$-value $1.43\times 10^{-3}$. In general, PartIES and CIMLR that use diffused kernels have better subtyping performance (in terms of associating with patient survival) than that without diffusion, while the performances of SNF with/without diffusion are very similar, consistent with observations in simulation results.

**Table 2:** TCGA BLCA, LIHC, and THCA cancer subtyping with (i) numbers of subtypes, and (ii) log-rank survival $P$-values

Methods		PartIES		CIMLR		SNF	
Diffusion		Yes	No	Yes	No	Yes	No
BLCA	number of clusters	6	6	4	4	4	4
	Survival $P$-values	$2.30\times 10^{-5}$	$3.76\times 10^{-4}$	$3.67\times 10^{-4}$	0.06	$7.39\times 10^{-3}$	$1.52\times 10^{-3}$
LIHC	number of clusters	4	4	3	3	2	2
	Survival $P$-values	$9.08\times 10^{-4}$	$3.69 \times 10^{-3}$	0.19	0.24	$7.81\times 10^{-3}$	$9.72\times 10^{-3}$
THCA	number of clusters	4	4	3	3	3	3
	Survival $P$-values	$1.43\times 10^{-3}$	0.11	0.67	0.58	0.93	0.56

### Individual cancer studies

#### The Cancer Genome Atlas bladder urothelial carcinoma

**Figure 4:**
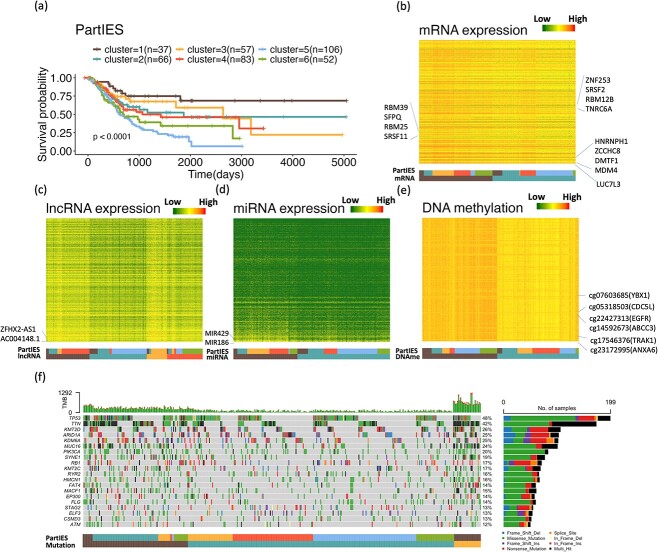
(a) Kaplan–Meier curves of the six TCGA BLCA subtypes by PartIES. (b)–(f) Heatmaps of top 500 features by KW test comparing feature measures across the six BLCA subtypes. (g) Mutation landscape of top 20 most frequently mutated genes across all BLCA tumors.

Studies have identified BLCA subtypes using omics data of TCGA BLCA tumors. For example, using mRNA expression data of 412 TCGA BLCA tumors, five subtypes were identified that are associated with patient survival with a $P$-value $4\times 10^{-4}$ [29]. Another study identified two major BLCA subtypes using mRNA expression, DNA methylation, DNA copy number, and somatic mutation data of 388 TCGA BLCA tumors with the iclusterBayes method. These two major subtypes are associated with patient survival with a $P$-value $4.2\times 10^{-4}$ [30]. Here we used mRNA, lncRNA, and miRNA expression data, DNA methylation data, and somatic mutation data of 401 TCGA BLCA tumors and identified six subtypes using the proposed PartIES with a survival $P$-value $2.30\times 10^{-5}$. Figure 4a presents Kaplan–Meier curves of the six BLCA subtypes by PartIES. We can see that subtype 5 with 106 tumors has the worst survival and a median survival time of 623 days. Subtype 1 with 37 tumors has the best survival where more than 50% of subjects were alive at the end of the follow-up.

**TCGA BLCA subtyping using a single omics data type vs. five omics data types**

**Figure 5:**
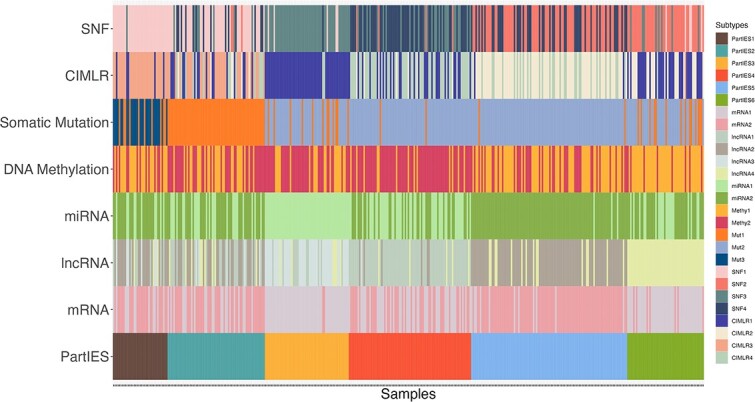
TCGA BLCA subtypes identified by PartIES and competing methods using five types of omics data vs. that using one type of omics data. Samples are ordered by PartIES subtypes.

Figure 4(b)–4(e) display heatmaps of top 500 features of mRNA, lncRNA, miRNA expression levels, and DNA methylation levels ranked by $P$-values from the Kruskal–Wallis (KW) test comparing feature levels across the six BLCA subtypes by PartIES. Figure 4f displays the heatmap of mutation profiles of top 20 most frequently mutated genes across 401 BLCA tumors. Subtypes identified by single omics data types with diffused kernels were also indicated, where two subtypes were identified using mRNA expression data, four using lncRNA expression data, two using miRNA expression data, two using DNA methylation data, and three using mutation data. This information was further displayed in Fig. 5.

We can see that overall, for the six BLCA subtypes by PartIES, mRNA and miRNA data provide very similar subtype structures and mainly separate PartIES subtypes 3 and 5. LncRNA data provide a distinct subtype structure that separates PartIES subtypes 1, 5 from 3, 4, and 6. Mutation data further separate PartIES subtypes 1, 2, and 5. DNA methylation data, on the other hand, do not provide clear subtype information. Only with all types of omics data, PartIES identifies current six BLCA subtypes.

When comparing the six PartIES subtypes to the four lncRNA subtypes, majority (85.5%) of tumors in PartIES subtype 4 are in lncRNA subtype 1, 84.9% of tumors in PartIES subtype 5 are in lncRNA subtype 2, 70.2% of tumors in PartIES subtype 3 are in lncRNA subtype 3, while all tumors in PartIES subtype 6 are in lncRNA subtype 4 (Fig. 4c). Using somatic mutation data, we further separate PartIES subtypes 1 and 2 from others. Although mRNA and miRNA expression data and DNA methylation data provide overlapping subtyping information as that of lncRNA expression data, only when we use all five types of omics data, we can identify current six BLCA subtypes. More details on single omics data subtyping results and important omics profiles that differentiate the six BLCA subtypes are included in the Supplementary Materials.

**Investigate subtype-differentiated important genes using the PPI network**

To investigate biological meanings of the six BLCA subtypes by PartIES, we examined omics features that differentiate them at a Bonferroni-adjusted KW test $P$-value threshold 0.01. From a collection of differentially expressed genes (mRNA, lncRNA, and miRNA genes), differentially methylated genes (CpGs were mapped to genes), and differentially mutated genes, we identified important genes that are highly interacting with others using protein-protein interaction (PPI) network [17] from the STRING database [31] where we kept edges having interaction scores $>0.4$ using Cytoscape (version 3.10.1) [32].

Details of mapping differential omics profiles to the PPI network are included in the Supplementary Materials. Briefly, we selected top 200 differential profiles from each omics data type and ended with a network with 455 genes and 1,054 edges after removing overlapping genes. Using the Largest Subnetwork app in Cytoscape, we partitioned this network into sub-networks such that nodes in the same sub-network are connected while those in different sub-networks are not, and worked with the largest sub-network with 328 genes.

We then used three metrics to measure interactions among the 328 genes: degree, stress, and betweenness centrality. Degree of a gene is the number of genes it connects. Stress of a gene is the number of shortest paths between two other genes that this gene passes. Betweenness centrality of a gene is the normalized ratio of the number of shortest paths between two other genes that this gene passes over all the shortest paths between the two genes. Table 3 displays top 10 genes ranked by each of the three metrics. Genes rank on top are potentially important BLCA genes. For instance, gene *HNRNPH1* has the highest mRNA expression levels in tumors in PartIES subtypes 3 (Fig. 4b), who had relatively good survival. Studies have suggested that low expression levels of *HNRNPH1* are associated with poor survival in BLCA [33]. Gene *CDC5L* (mapped from CpG cg05318503) has relatively high methylation levels in PartIES subtype 6 tumors and low levels in subtype 4 tumors (Fig. 4e), which has been found to be related to the apoptosis and migration of bladder cancer cells [34]. Gene *YBX1* (mapped from CpG cg07603685) has relatively high methylation levels in PartIES subtype 6 tumors and low levels in subtype 4 tumors (Fig. 4e) and has been found to be associated with bladder cancer progression [35].

**Table 3:** Top 10 genes ranked by degree, stress, and betweenness centrality

Top 10 Genes	Degree	Top 10 Genes	Stress	Top 10 Genes	Betweenness Centrality
RBM39	45	EGFR	129 808	EGFR	0.290
RBM25	38	DHX9	64 208	DHX9	0.101
HNRNPH1	36	HNRNPH1	50 206	YBX1	0.077
SRSF2	36	YBX1	47 262	HNRNPH1	0.076
EGFR	35	RBM39	41 542	NF1	0.063
PRPF8	35	SRSF2	37 924	PRPF8	0.060
CDC5L	35	CDC5L	36 762	TNRC6A	0.057
DHX9	35	PRPF8	36 374	RBM39	0.056
LUC7L3	33	TNRC6A	35 366	SRSF2	0.056
SRSF11	33	SFPQ	30 360	RUNX1	0.055

**Pathway activities of the six BLCA subtypes by PartIES**

**Figure 6:**
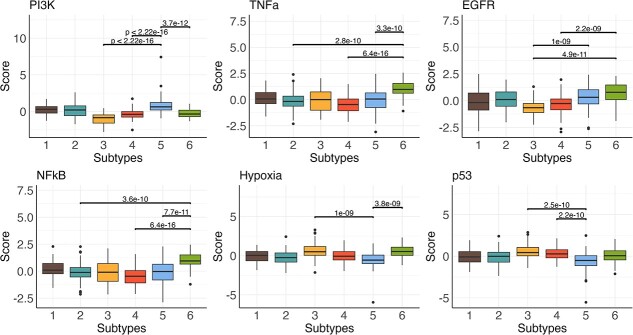
Boxplot of top 6 cancer-related pathways’ activity scores ranked by KW test comparing scores across the six BLCA subtypes by PartIES. Also displayed are the $P$-values of the three pairs of subtypes with the most significant difference from the Wilcoxon rank sum test comparing pathway activities between each pair of subtypes.

We further compared pathway activities across the 6 BLCA subtypes of 11 cancer-related pathways (EGFR, MAPK, PI3K, VEGF, JAK-STAT, TGFb, TNFa, NFkB, Hypoxia, p53-mediated DNA damage response, and Trail (apoptosis)) calculated using mRNA expression levels with the R package ‘PROGENy’ [36]. PROGENy (Pathway RespOnsive GENes) assigns pathway scores to tumors using the fitted coefficients matrix and tumors’ mRNA expression levels. We compared pathway activity scores across the six BLCA subtypes using the KW test where all 11 pathways are significant after Bonferroni correction. We examined top 6 most significant pathways (Fig. 6). We can see that tumors in PartIES subtype 5, which have the worst survival, have the highest PI3K activity scores but the lowest p53 activity scores. Hyper-activations of the PI3K pathway was linked to various malignant cancer progresses such as tumor cell proliferation, metastasis, and drug resistance [37]. Accumulations of p53 protein is important to suppress cancer development and abnormal changes in p53 pathway activities potentially due to TP53 gene mutation are significantly associated with poor patient survival [38]. Moreover, tumors in PartIES subtypes 5 and 6 with worse survival have relatively high EGFR activities, which is an indicator of more aggressive cancer behavior and poor survival outcomes in bladder cancer [39].

#### The Cancer Genome Atlas liver hepatocellular carcinoma

Much research has been done to identify liver cancer subtypes. TCGA research network identified three liver cancer subtypes using copy number variants, DNA methylation, mRNA expression, miRNA expression, and reverse-phase protein array data of 183 TCGA LIHC tumors with iCluster [40]. The three subtypes are not significantly associated with patient survival. We previously developed abSNF [41] to integrate mRNA expression, DNA methylation, and somatic mutation data of 161 TCGA LIHC tumors and identified five subtypes that are associated with patient survival with a $P$-value 0.046. Here PartIES used mRNA, lncRNA, miRNA expression data, DNA methylation data, and somatic mutation data of 362 TCGA LIHC tumors and identified four subtypes that are associated with patient survival with a $P$-value $9.08\times 10^{-4}$. We similarly investigated biological meanings of the four LIHC subtypes with details in the Supplementary Materials and Table S1, Figs S5–7.

#### The Cancer Genome Atlas thyroid carcinoma

Using 496 TCGA THCA tumors, TCGA research network identified two major subtypes based on driver mutations with no patient survival analysis being done. Here PartIES used mRNA, lncRNA, miRNA expression data, DNA methylation data, and somatic mutation data of 484 TCGA THCA tumors and identified four subtypes that are significantly associated with patient survival with a $P$-value $ 1.43 \times 10^{-3}$. We similarly investigated biological meanings of the four THCA subtypes with details in the Supplementary Materials and Table S2, Figs S8–10.

#### Sensitivity analysis

We conducted sensitivity analyses using subsets of the five omics data types. We examined PartIES and competing methods when removing data types with (1) similar clustering structures, and (2) distinct clustering structures. For example, from Fig. 5 for BLCA, we see that lncRNA and somatic mutation data provide distinct clustering structures, while mRNA expression, miRNA expression and DNA methylation data provide similar cluster information. Therefore, we repeated the analyses removing (i) mRNA data, (ii) mRNA and DNA methylation data, (iii) mutation data, and (iv) mutation and lncRNA data. Table 4 displays these BLCA subtyping results. When removing data types with similar structures, all methods have similar clustering results as that using five omics data types where PartIES performs the best in terms of patient survival (Table 4, Analysis 1–3). When removing data types with distinct cluster structures, all methods have worse clustering results than that using all five omics data types (Analysis 4–5) as expected. Similar patterns were observed for LIHC and THCA with details in the Supplementary Materials Tables S3–4.

**Table 4:** Sensitivity analysis of TCGA BLCA subtyping with different omics data types

**Cancer**	**Analysis**	**Omics Data types**		**PartIES**	**CIMLR**	**SNF**
BLCA	1	LncRNA, mutation, miRNA, DNA methylation, mRNA	Number of clusters	6	4	4
			Survival $P$-value	2.30E-05	3.67E-04	7.39E-03
	2	LncRNA, mutation, miRNA, DNA methylation	Number of clusters	6	4	4
			Survival $P$-value	1.09E-05	2.61E-03	3.04E-03
	3	LncRNA, mutation, miRNA	Number of clusters	6	3	4
			Survival $P$-value	3.19E-05	4.75E-04	3.09E-03
	4	LncRNA, miRNA, DNA methylation, mRNA	Number of clusters	5	3	3
			Survival $P$-value	3.17E-03	0.030	0.038
	5	miRNA, DNA methylation, mRNA	Number of clusters	4	3	3
			Survival $P$-value	0.18	0.04	0.11

## Discussion

In this paper, we developed PartIES, a Partition-level Integration framework that uses diffusion-Enhanced Similarities to preserve data-type-specific cluster structures for a more accurate clustering result using multi-omics data. PartIES denoises individual similarity matrices through diffusion and partitions denoised similarity matrices before integrating them iteratively.

Simulation studies suggested a much-improved cluster accuracy with the proposed diffusion step as a general strategy, especially on the competing method CIMLR, which only uses global similarity in the original algorithm. SNF has only granular improvement with diffusion because local similarities were applied in fusing multiple similarity matrices in SNF. For overall clustering performance, PartIES performs the best under settings when three data types provide different subtype structures, the scenario PartIES was designed for. Under settings when three data types provide the same subtype structures, PartIES performs similarly as CIMLR, with both better than SNF.

We applied PartIES and competing methods to identify BLCA, LIHC, and THCA subtypes using mRNAs, lncRNAs, and miRNAs expression data, DNA methylation data, and somatic mutation data from TCGA. The six BLCA subtypes, the four LIHC subtypes, and the four THCA subtypes by PartIES can most significantly differentiate patient survival across all methods. More importantly, some omics data types provide different subtype structures from others. When integrating data-type-specific partition level information, we can preserve distinct subtype structures and more accurately subtype all tumors. Further investigations on biological meanings of the identified subtypes suggest that among subtype-associated genes, many of those that are highly interacting with other genes are known important cancer genes. The identified cancer subtypes also have different activity levels for some known cancer-related pathways.

One limitation of PartIES is that we need to determine numbers of subtypes using individual omics data types. There are currently no universal rules to select the number of clusters. We used the eigengap criterion to guide our selections. While being a limitation, eigengap can also help determine if a data type is informative for clustering. We conducted additional simulation studies and showed that when no cluster is in a data type, eigengaps do not change with different numbers of clusters (see Supplementary Materials). A perturbation method was recently developed [21] to determine the number of clusters. For further work, we will explore using perturbations with spectral clustering to determine the number of clusters. Another limitation is that we only use counts of non-synonymous mutations per gene across the genome as a sample’s mutation profile, which ignores mutation types. With the current TCGA tumor sample sizes, only few genes have more than one mutations per gene. We will explore meta-analysis with a much larger sample size to allow finer analyses with mutation types for future work. We also want to mention that PartIES is computationally efficient and uses comparable computational time as that of SNF and CIMLR. Details are in the Supplementary Materials Table S6.

**Key Points**

- The proposed PartIES integrate partition level information iteratively from individual types of omics data to better preserve distinct data-type-specific cluster structures.
- The proposed PartIES uses a diffusion step to denoise individual similarity matricies.
- The proposed PartIES has the best clustering performance when different types of omics data provide distinct clustering structures or provide with similar structure but different effect sizes, and performs similarly as competing methods when different types of omics data provide similar clustering structures.

## Supplementary Material

PartIES_Oxford_suppl_bbae609
