# Supplementary material for: PartIES: a disease subtyping framework with Partition-level Integration using diffusion-Enhanced Similarities from multi-omics Data
Source: Brief Bioinform. 2024 Nov 25;26(1):bbae609. doi: 10.1093/bib/bbae609 (PMC11586768; doi:10.1093/bib/bbae609)
Supplement: PartIES_Oxford_suppl_bbae609 [file parties_oxford_suppl_bbae609.pdf]

Supplementary Data for “PartIES: a disease subtyping framework with Partition-level Integration using diffusion-Enhanced Similarities from multi-omics Data”

Yuqi Miao, Huang Xu, Shuang Wang

October 16, 2024

A PartIES supplements

A.1 Optimization details of $Z_s, s = 1, \cdots, S$

In Step 1 of the optimization procedure, we fix F_s and optimize $Z_s, s = 1, \cdots, S$ separately for each data type s as follows:

$$\min_{Z_s} \left\{ < D_{K_{s,enh}}, Z_s >_F + \beta ||Z_s||_F^2 + \gamma tr(F_s^T (I - Z_s) F_s) \right\} s.t. \sum_{j=1}^{n} Z_s(i,j) = 1, Z_s(i,j) \geq 0. \quad (1)$$

Specifically, we assumed subjects are independent, thus we can perform row-wise optimization for Z_s. For i^{th} row, i.e., for the ith subject, the optimization is a quadratic convex optimization. We can rewrite formula (1) as follows:

$$\begin{aligned} &\min_{Z_s(i,\cdot)} \sum_{j=1}^{n} \left\{ \beta Z_s(i,j)^2 + \left(D_{K_{s,enh}}(i,j) + \frac{\gamma}{2} ||F_s(i,\cdot) - F_s(j,\cdot)||_F^2 \right) Z_s(i,j) \right\} \\ &s.t. \sum_{j=1}^{n} Z_s(i,j) = 1, Z_s(i,j) \geq 0. \end{aligned} \quad (2)$$

To optimize each row $Z_s(i,)$ for data type s, for simplicity, we omit the subject index i and data type index s and express $a_j = Z_s(i,j)$ and $v_j = -\frac{1}{2\beta} \left(D_{K_{s,enh}}(i,j) + \frac{\gamma}{2} ||F_s(i,\cdot) - F_s(j,\cdot)||_F^2 \right)$. The optimization of row i of $Z_s(i,\cdot)$ for data type s becomes:

$$\min_{\tilde{a}} \sum_{j=1}^{n} \left(a_j^2 - 2v_j a_j \right) \; s.t. \sum_{j=1}^{n} a_j = 1, a_j \geq 0. \quad (3)$$

This can be further simplified into a quadratic optimization problem as:

$$\min_{\tilde{a}} \frac{1}{2} \|\tilde{a} - \tilde{v}\|_F^2 \; s.t. \sum_{j=1}^{n} a_j = 1, a_j \geq 0.$$

We can solve the optimization using the Lagrangian approach with parameter λ and slack variables $\eta_{n \times 1} \geq 0$:

$$\mathcal{L}(\tilde{a}, \lambda, \eta) = \frac{1}{2} \|\tilde{a} - \tilde{v}\|_F^2 + \lambda(\tilde{a}^T \mathbf{1} - 1) - \eta^T \tilde{a}.$$

To set up the KKT condition, for $j = 1, \cdots, n$:

$$\begin{aligned} a_j - v_j + \lambda - \eta_j &= 0 \\ \sum_{j=1}^{n} a_j - 1 &= 0 \\ a_j &\geq 0 \\ \eta_j &\geq 0 \\ a_j \eta_j &= 0. \end{aligned}$$

By eliminating the slack variables (using standard techniques in [1]), we can further simplify the conditions as:

$$a_j = (\mu_j - \eta^*)_+$$
$$\eta_j = (\eta^* - \mu_j)_+, j = 1, \cdots, n,$$

where $\mu_j = v_j - \frac{1}{n}\sum_{j=1}^{n} v_j + \frac{1}{n}$, $\eta^* = \frac{1}{n}\sum_{j=1}^{n} \eta_j$ and $(\cdot)_+ = \max(\cdot, 0)$. Finally, we obtain the solution to the equation system above by solving the following equation:

$$f(\eta^*) = \frac{1}{N-1}\sum_{j=1}^{N-1}(\eta^* - \mu_j)_+ - \eta^* = 0. \tag{4}$$

Details of setting β and γ For each data type s, we set β to regularize each row of $Z_{s,n\times n}$ to have the k largest measures to be greater than 0, and the other measures to be close to zero, i.e., to use local information. A similar strategy has been used by [2, 3]. Specifically, according to the KKT condition, the optimal solution for $Z_s(i,j)$ can be expressed as:

$$Z_s(i,j) = (\lambda^* - \frac{d_s^2(i,j)}{2\beta})_+, \tag{5}$$

where $d_s^2(i,j) = (D_{K_{s,enh}}(i,j) + \frac{\gamma}{2}\|F_s(i,\cdot) - F_s(j,\cdot)\|_F^2)$. To achieve desired similarity measures for subject i where only the top k largest similarities are kept as non-zero, we should approximately achieve $\frac{d_s(i,(k))^2}{2\beta} \leq \lambda^* \leq \frac{d_s(i,(k+1))^2}{2\beta}$, where $d_s(i,(j))$ is the distance between subject i and his/her j^{th} nearest neighbor. Due to the constraint $\sum_{j=1}^{n} Z_s(i,j) = 1$, we have:

$$k\lambda^* - \sum_{j=1}^{k}\frac{d_s^2(i,(j))}{2\beta} = 1, \tag{6}$$

which leads to the following inequity:

$$k\frac{d_s^2(i,(k+1))}{2\beta} - \sum_{j=1}^{k}\frac{d_s^2(i,(j))}{2\beta} \geq 1. \tag{7}$$

If we set inequality (7) as equality, we can get an approximate value of β for subject i as

$$\beta_{i,s} = k\frac{d_s^2(i,(k+1))}{2} - \sum_{j=1}^{k}\frac{d_s^2(i,(j))}{2}, \tag{8}$$

which is the sum of the gap between the distance of subject i to his/her $(k+1)^{th}$ nearest neighbor and to his/her k nearest neighbors. Under this rationale, we set the hyper-parameter β_s for data type s as the average enhanced kernel distance gaps of all subjects and set β_s as follows:

$$\beta_s = \frac{1}{2n}\sum_{i=1}^{n}\sum_{j=1}^{k}(D_{K_{s,enh}}(i,(k+1)) - D_{K_{s,enh}}(i,(j))),$$

where n is the number of subjects, $D_{K_{s,enh}}(i,(j))$ is the enhanced kernel distance between subject i and his/her j^{th} nearest neighbor using data type s. We set $\gamma = \frac{1}{S}\sum_{s=1}^{S}\beta_s$. We don't update the

hyper-parameters with optimization as the hyper-parameters change trivially among iterations.

A.2 To use eigengap criterion to guide the selection of numbers of clusters

To conduct spectral clustering on a symmetric, positive definite similarity matrix $S_{n\times n}$, it uses normalized graph Laplacian $L = I - D^{-1/2}SD^{-1/2}$, where $D_{n\times n} = \text{diag}(\sum_{j=1}^{n} S_{ij}, i = 1, \cdots, n)$ is the degree matrix. If we denote the ith smallest eigenvalue of L as λ_i, the ith eigengap E_i is defined as $E_i = \lambda_{i+1} - \lambda_i, i \in \{1, \cdots, n-1\}$. The number of clusters c can be guided by $c = \text{argmax}_i E_i$ together with a plot of eigengap.

In simulation studies, we used the true number of clusters C for the two competing methods, SNF and CIMLR. For the proposed PartIES, we used the eigengap criterion to guide the choice of numbers of clusters on initialized Z_s for individual data types $C_s, s = 1, \cdots, S$ and used the true number of clusters C when integrating multiple omics data types for clustering.

In real data applications, we selected the number of clusters C for each comparing method separately, guided by the eigengap criterion. For the proposed PartIES, the number of clusters for each single data type C_s is guided by the eigengap of the initialized Z_s, and the number of clusters C when integrating multiple omics data types is guided by the eigengap of $\sum_{s=1}^{S} w_s 2F_s F_s^T$ using initialized individual partition information F_s.

Eigengap can also help determine if a data type is informative for clustering. To demonstrate this, we conducted a simulation study where we simulated 3 omics data types each with 10,000 features for 200 samples. The first omics data type is pure noise with no signal features. The second omics data type has 100 signal features out of the 10,000 features with 100 samples being generated from N(1,1) and the rest 100 samples being generated from N(2,1). The third omics data type has 200 signal features and were generated similarly as the second omics data type. We simulated 100 datasets. We similarly used eigengaps to decide number of clusters identified by each data type and included results in Figure S1 below. We can see that for the first omics data type with pure noise, eigengaps do not change across different numbers of clusters, while the other two data types with cluster information, the largest eigengap was achieved when the number of clusters is 2. These results suggest that eigengap is able to inform us if there is clustering information in a data type.

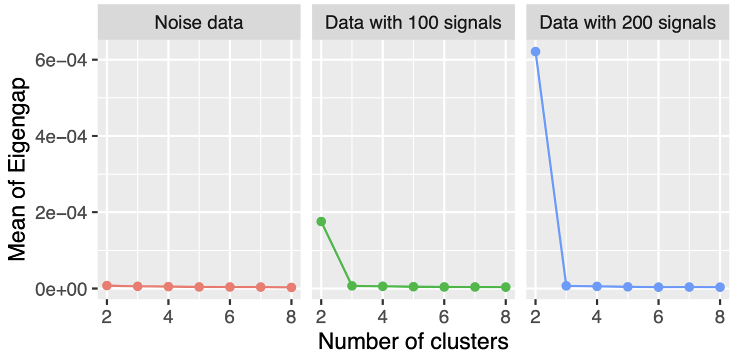

Figure S1: Means of eigengaps of different number of clusters across 100 simulations

A.3 Choices of number of neighbors k

In order to check if PartIES and competing methods are sensitive to the choices of number of neighbors k, we conducted simulation studies using three different k's, $k = \{n/3, n/4, n/5\}$, where n is the number of samples and is set at 200 for this simulation study. As shown in Figure S2, all three methods are not very sensitive to the choices of number of neighbors k, while PartIES is less affected than CIMLR and SNF.

B Additional simulation studies with omics data from normal and non-normal distributions and a smaller sample size

We conducted additional simulation studies to evaluate the performance of PartIES and competing methods when 1) features are generated from Bernoulli distributions and normal distributions, and ii) when sample size is small.

B.1 Simulation settings

We simulated all omics data types using normal distributions in the main text for simplicity. We conducted additional more realistic simulations. Specifically, we considered one type of omics data to be binary to mimic mutation presence/absence per gene and the other two types of omics data to be continuous to mimic gene expression data. We simulate 3 types of omics data for 200 patients with four clusters, each with 50 patients. Each data type has 10,000 features, including both signal and noise features. The number of signal features in the three data types are the same, ranging from 10 to 200 with a grid of 10, and signal features within one data type have the same effect size. The first data type is the gene mutation presence/absence data. For signal features, mutation occurrence per gene was generated from a Bernoulli distribution with different probabilities of success for samples in different clusters. For noise features, mutation occurrence per gene was generated from a Bernoulli(0.1) distribution for all samples. The other two data types are gene expression data, where signal features were generated from normal distributions with different means and standard deviation 1 for samples from different clusters. Noise features were generated from N(0,1) for all samples. We similarly considered three simulation settings as in the main text.

In simulation setting I, three data types provide the same clustering structures with signal features in different data types have similar effect sizes. Specifically, signal features in data type 1 are generated from Bernoulli distribution with probability of success equal to $0.1, 0.3, 0.7, 0.9$ for samples in 4 clusters, separately; signal features in data type 2 are generated from normal distribution with mean equal to $0.5, 1.2, 1.5, 2$ for 4 clusters, separately; and signal features in data type 3 are generated from normal distribution with mean equal to $0.5, 1, 1.3, 2$ for 4 clusters, separately.

In simulation setting II, three data types provide similar clustering structures but signal features in different data types have different effect sizes. Specifically, signal features in data type 1 are generated from Bernoulli distribution with probability of success equal to $0.1, 0.3, 0.7, 0.9$ for 4

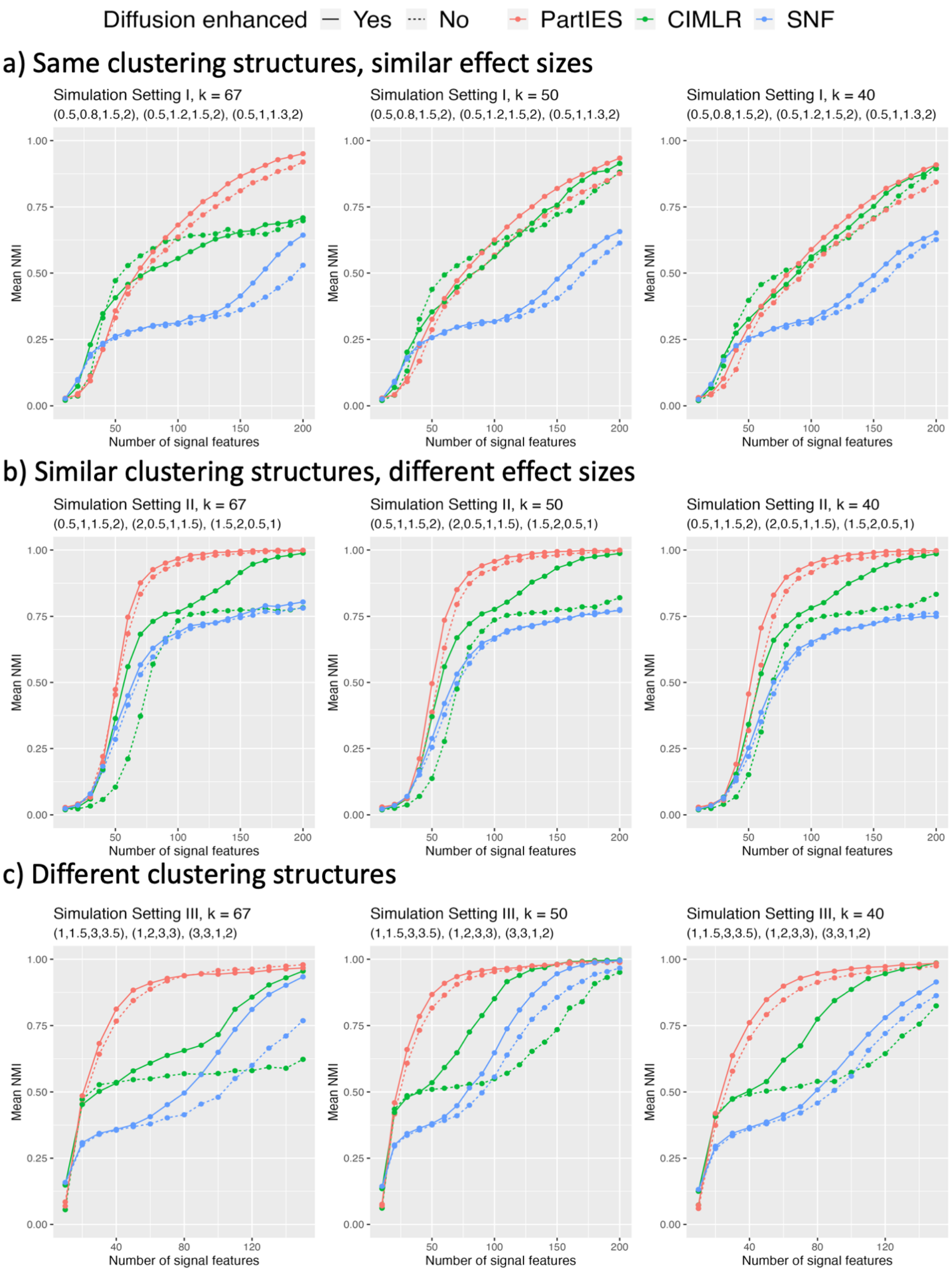

Figure S2: Clustering performance of PartIES and competing methods with different choices of number of neighbors k. Displayed are mean NMIs across 1,000 simulations.

clusters, separately, i.e., data type 1 separates all 4 clusters while it separates clusters 1 and 4 the best. Signal features in data type 2 are generated from normal distribution with mean equal to $2, 0.5, 1, 1.5$ for 4 clusters, separately, i.e., data type 2 separates clusters 1 and 2 the best. signal features in data type 3 are generated from normal distribution with mean equal to $1.5, 2, 0.5, 1$ for

4 clusters, separately, i.e., data type 3 separates clusters 2 and 3 the best. PartIES would perform well in this setting.

In simulation setting III, three data types provide different cluster structures. Specifically, signal features in data type 1 mainly separate clusters 1 and 2 from clusters 3 and 4 with probability of success in 4 clusters equal to $0.1, 0.2, 0.7, 0.8$, separately. Signal features in data type 2 mainly separate clusters 1 and 2 from clusters 3 and 4, and can further separate cluster 1 and cluster 2 with means in 4 clusters being $1, 2, 3, 3$, separately. Signal features in data type 3 separate clusters 1 and 2 from clusters 3 and 4, and can further separate cluster 3 and cluster 4 with means in 4 clusters being $3, 3, 1, 2$, separately. This is the setting the proposed PartIES is designed for.

We also considered a much smaller sample size with 60 subjects and 4 equal-sized clusters, each with 15 subjects to evaluate the performance of PartIES and competing methods. We similarly considered three simulation settings as above.

B.2 Additional simulation results

Figure S3-S4 display the simulation results when n=200 and n=60, separately. We can see that when sample size is relatively large (Figure S3), the performance of PartIES and competing methods when integrating binary and normal data types is similar with that when integrating all normal data types (Figure 3). When sample size is relatively small (Figure S4), For simulation setting III, PartIES has much better performance when effect sizes are not too small (with more than 50 signal features), and when effect size is small, all methods have comparable performance. For simulation settings I and II, PartIES has the best performance when effect size is much bigger with more than 100 signal features. When effect size is small with number of signal features smaller than 100, PartIES has comparable performance with SNF, while CIMLR has the best performance. This is because CIMLR can better aggregate small effect sizes when there are common cluster structures across different data types.

C TCGA applications supplements

C.1 Individual cancer case studies

C.1.1 TCGA BLCA

Detailed comparisons between BLCA subtypes using single omics data type v.s. five omics data types

In the main text, we described how individual omics data types help separate the six subtypes by PartIES. In this section, we compared subtypes by PartIES and subtypes using one type of omics data in detail. We also discussed important molecular features that differentiate the six BLCA subtypes.

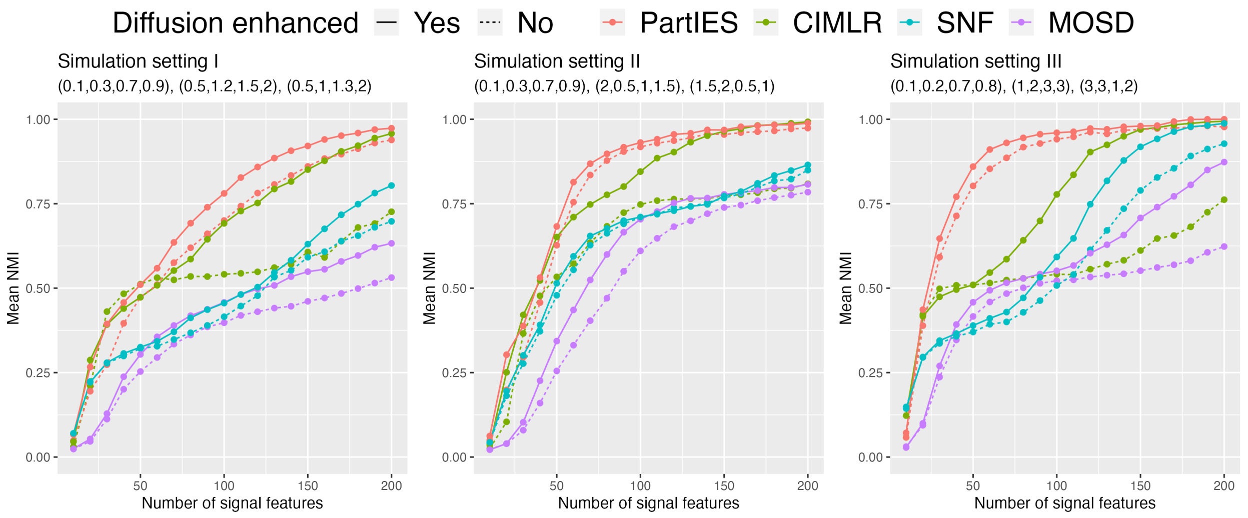

Figure S3: Mean NMI of PartIES and comparing methods across 100 simulations for N=200 patients using the three simulation settings considered when omics data were generated from Bernoulli distributions and normal distributions

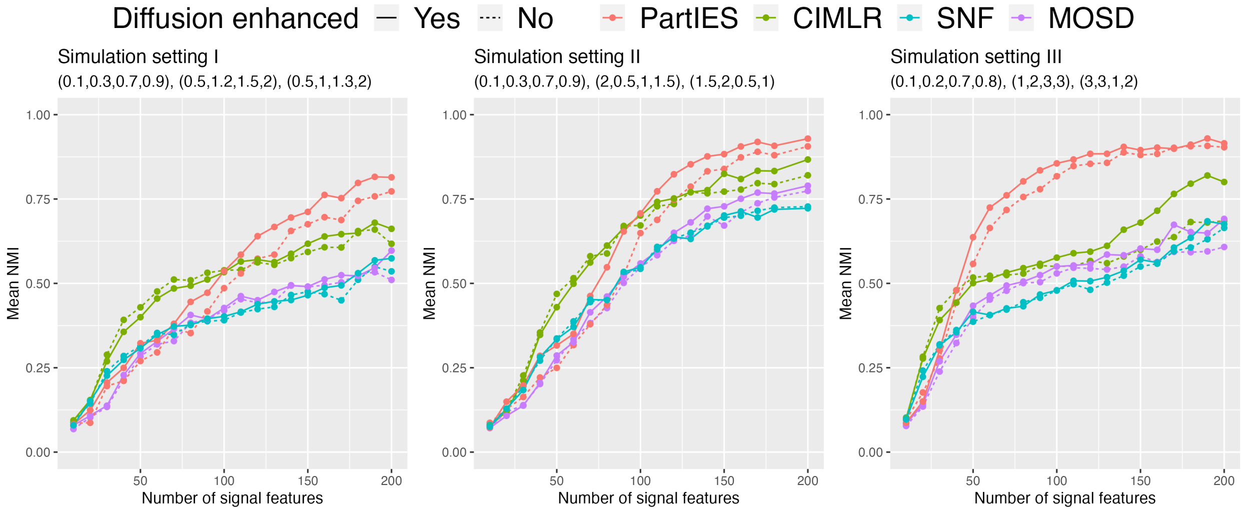

Figure S4: Mean NMI of PartIES and comparing methods across 100 simulations for N=60 patients using the three simulation settings considered when omics data were generated from Bernoulli distributions and normal distributions

When comparing the two subtypes by mRNA expression data to the 6 subtypes by PartIES, we can see that mRNA expression data can separate PartIES subtypes 3 and 6 from subtypes 1, 2, and 5, while PartIES subtype 4 is divided almost equally into the two mRNA subtypes (Figure 5). More specifically, most tumors in PartIES subtypes 3 (96.5%) and 6 (86.5%) are in the mRNA subtype 1, while the majority of tumors in PartIES subtypes 1 (67.6%), 2 (66.7%), and 5 (89.6%) are in the mRNA subtype 2. Several known important BLCA genes, such as genes *MDM4*, *DMTF1*, and *ZCCHC8* have significantly different expression levels across the 6 subtypes by PartIES where tumors in subtypes 3 and 6 have relatively high expression levels (Figure 4b). Studies have suggested that over-expression of *MDM4* gene might lead to the escape of p53-dependent growth control

in bladder cancer cells [4], and over-expression of *DMTF1* gene suppresses bladder cancer tumor progression [5], while *ZCCHC8* gene was identified as a potential antigen for mRNA-based vaccines against BLCA [6].

When comparing the 6 PartIES subtypes to the 4 lncRNA subtypes, we notice that lncRNA expression data can separate PartIES subtypes 1 and 5 from subtype 4, from subtype 3, and from subtype 6 (Figure 5). More specifically, majority of tumors in PartIES subtype 5 (84.9%) and subtype 1 (56.8%) are in lncRNA subtype 2, majority (85.5%) of tumors in PartIES subtype 4 are in lncRNA subtype 1, 70.2% of tumors in PartIES subtype 3 are in lncRNA subtype 3, and all tumors in PartIES subtype 6 are in lncRNA subtype 4. Several important lncRNAs such as ZFHX2-AS1 and AC004148.1 have different expression levels across the 6 PartIES subtypes when tumors in subtype 6 have relatively low expression levels and tumors in subtypes 3 and 4 have relatively high expression levels (Figure 4c). Low expression levels of these two lncRNAs have been shown to be associated with worse survival [7, 8].

The two subtypes from miRNA expression data mainly help separate PartIES subtype 3 from subtype 5 (Figure 5). Specifically, all tumors in PartIES subtype 3 are in miRNA subtype 1, while 97.2% tumors in PartIES subtype 5 are in miRNA subtype 2. Several important BLCA miRNAs such as MIR429 and MIR186 display relatively low expression levels in PartIES subtype 5 but relatively high expression levels in PartIES subtype 3 (Figure 4d). High expression level of MIR429 has been shown to be associated with better survival in BLCA patients [9], and high expression of MIR186 suppresses bladder cancer cell proliferation and metastasis [10].

Using DNA methylation data, two subtypes were identified. Majority of tumor samples in PartIES subtypes 5 (61.3%) and 6 (84.6%), which have worse survival than other PartIES subtypes, are in the methylation subtype 1, while the majority of tumor samples in PartIES subtypes 3 (66.7%) and 4 (84.3%), who have relatively good survival, are in DNA methylation subtype 2. Several DNA methylation sites that are differentially methylated across 6 PartIES subtypes are near important BLCA cancer genes, e.g., cg23172995 is near *ANXA6* gene and cg17546376 is near *TRAK1* gene [11, 12].

Three subtypes were identified using somatic mutation data, which helped separate PartIES subtypes 1 and 2 from other subtypes. Specifically, all tumors in PartIES subtype 2 are in mutation subtype 1, and 73.0% of tumors in PartIES subtype 1 are in mutation subtype 3. We notice that tumors in PartIES subtype 1, which have the best survival, have the highest mutation burden, and tumors in PartIES subtype 2, which have the 2nd best survival, also have a relatively high mutation burden. This observation is consistent with previous findings [13]. Mutation frequencies of important cancer genes varied greatly among the six PartIES subtypes. For example, 43.2% and 27.3% of tumor samples in PartIES subtypes 1 and 2 have *PIK3CA* mutations, respectively, while only 10%-20% of tumor samples in other PartIES subtypes have *PIK3CA* mutations.

Mapping BLCA subtype differential molecular features on the PPI network

We used a Bonferroni-adjusted P-value threshold 0.01 for the Kruskal-Wallis (KW) test comparing molecular features across the six BLCA subtypes by PartIES. There are 12,742 differentially expressed mRNAs, 5,173 lncRNAs, 127 miRNAs, 44,483 differentially methylated CpG sites, and 96 differentially mutated genes. We selected top 200 differentially expressed mRNAs (mapped to 197

genes in the PPI network), top 200 lncRNAs (mapped to 1 gene in the PPI network), and top 200 differentially methylated CpG sites (mapped to 166 genes in the PPI network). We used all 127 differentially expressed miRNAs (no genes mapped in the PPI network) and all 96 differentially mutated genes (mapped all 96 genes in the PPI network). There are 455 genes with 1,054 edges mapped to the PPI network after removing the overlapping genes.

C.1.2 TCGA LIHC

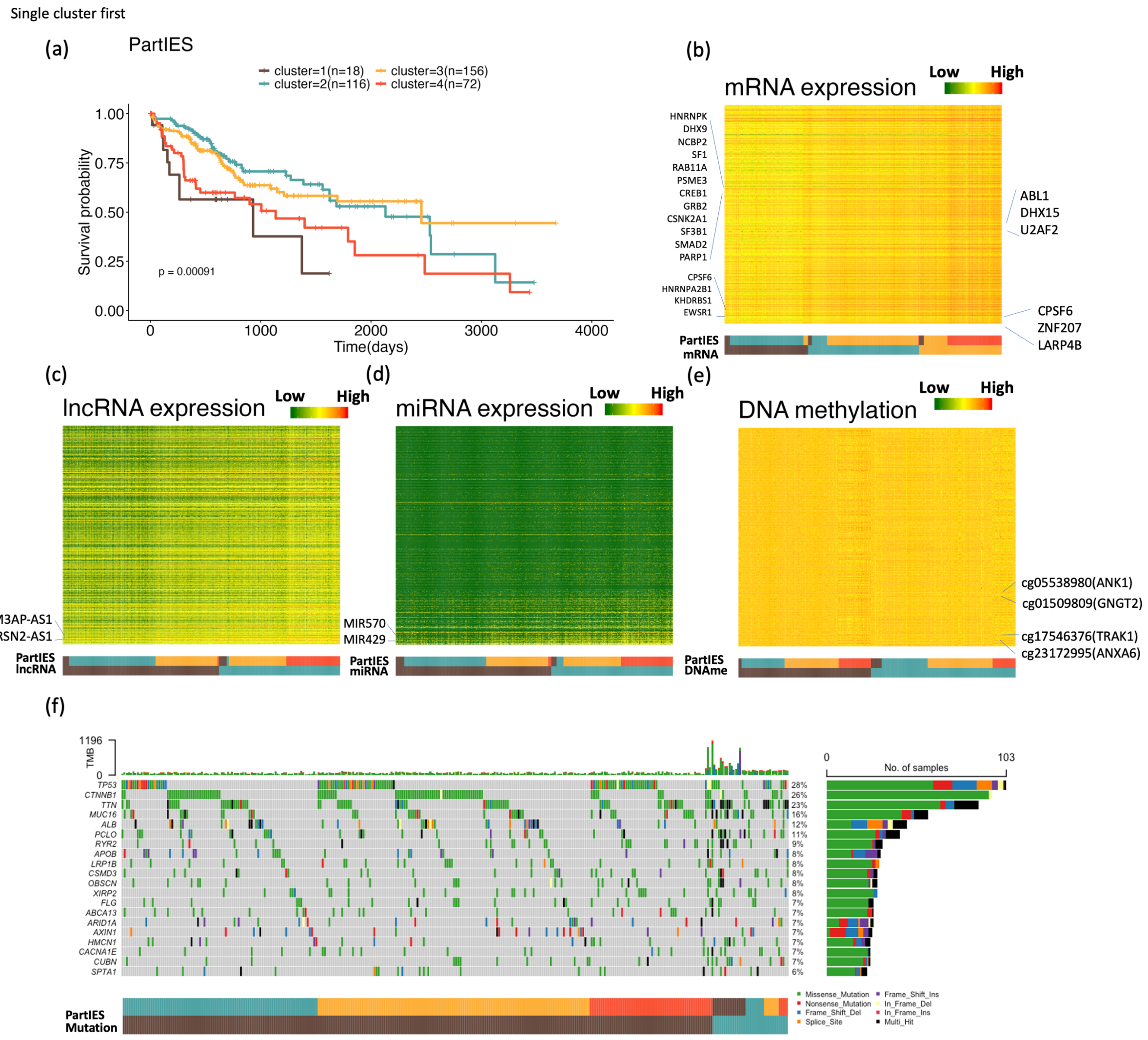

Figure S5: (a) Kaplan-Meier curves of the 4 TCGA LIHC subtypes by PartIES. (b-e) Heatmaps of top 500 features by KW test comparing feature measures across the 4 LIHC subtypes. (f) Mutation landscape of top 20 most frequently mutated genes across all LIHC tumors.

In the main text, we briefly reviewed current research on liver cancer subtyping using TCGA multi-omics data and showed that LIHC subtypes identified by PartIES can most significantly differentiate

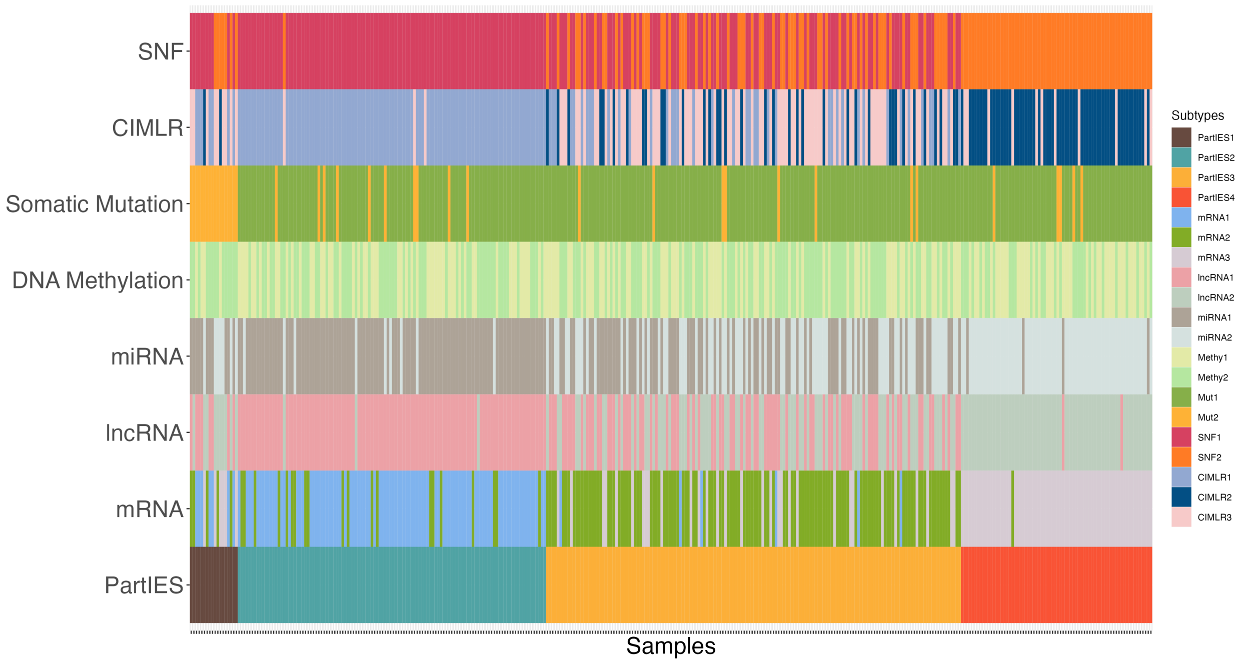

Figure S6: TCGA LIHC subtypes identified by PartIES and competing methods using five types of omics data vs. that using one type of omics data. Samples are ordered by PartIES subtypes.

patient survival across all comparing methods (Table 2). Specifically, PartIES identified 4 LIHC subtypes that are associated with patient survival with a P-value 9.08×10^{-4} using 362 TCGA LIHC tumors (Table 2). Figure S5a presents Kaplan-Meier curves of the 4 LIHC subtypes identified by PartIES. Subtype 1 containing 18 tumors has the worst survival with a median survival time of 931 days. Subtype 3 containing 156 tumors has the best survival with a median survival time of 2,456 days. In the following sections, we first compared LIHC subtypes identified by PartIES using five omics data types to those using one type of omics data. We then explored the biological meanings of the 4 PartIES LIHC subtypes by finding important subtype-differentiated LIHC genes on the PPI network and comparing cancer-related pathway activities across LIHC subtypes.

TCGA LIHC subtyping using a single omics data type vs. five omics data types

Figure S5b-S5e display the heatmaps of top 500 features in mRNA, lncRNA, miRNA expression levels, and DNA methylation levels ranked by P-values of Kruskal-Wallis (KW) test comparing features across the 4 LIHC subtypes by PartIES. Figure S5f displays the heatmap of mutation profiles of the top 20 most frequently mutated genes across all 362 LIHC tumors. Subtypes identified by individual omics data types using spectral clustering were also indicated. We note that 3 subtypes were identified using mRNA expression data only, 2 were identified using lncRNA expression data only, 2 were identified using miRNA expression data only, 2 were identified using DNA methylation data only, and 2 were identified using mutation data only. This information was further displayed in Figure S6.

Overall, mRNA expression data provides distinct structure information to separate subtypes 2, 3, and 4 by PartIES, while lncRNA and miRNA, which provide similar subtype information, help to further separate subtype 2 from subtype 4 by PartIES. DNA methylation data distinguishes

subtype 1 from partial of the 3 other subtypes by PartIES, while somatic mutation data further separates subtype 1 from the others. Specific details of subtype comparisons and important LIHC features differentiated the 4 PartIES LIHC subtypes are provided below.

The mRNA expression data helps to separate PartIES subtypes 2, 3, and 4. Specifically, most tumors (82.8%) in PartIES subtype 2 are in the mRNA subtype 1, most tumors (76.3 %) in PartIES subtype 3 are in the mRNA subtype 2, and most tumors (98.6%) in PartIES subtype 4 are in the mRNA subtypes 3 (Figure S6). Important LIHC genes show significantly different expression patterns among the 4 LIHC subtypes by PartIES. For example, tumors in PartIES subtype 4 have relatively high expression levels of genes *LARP4B*, *ZNF207*, and *CPSF6* (Figure S5b). High expression level of gene *LARP4B* in liver cancer patients is associated with shorter overall/relapse-free survival [14], and high expression of level gene *CPSF6* has also been shown to be related to poor survival [15], while gene *ZNF207* was identified as an immunosuppressive target of liver cancer for immunotherapy development [16].

LncRNA and miRNA expression data can similarly separate tumors in PartIES subtype 2 from subtype 4 by PartIES. Specifically, 97.4% (and 92.2%) of tumors in PartIES subtype 2 are in the lncRNA subtype 1 (and miRNA subtype 1), while 97.2% (and 94.4%) of tumors in PartIES subtype 4 are in the lncRNA subtype 2 (and miRNA subtype 2) (Figure S6). Tumors in PartIES subtype 4 have relatively high expression levels at important LIHC lncRNAs such as NRSN2-AS1 and MCM3AP-AS1 (Figure S5c) and important LIHC miRNAs such as MIR429 and MIR570 (Figure S5d). Notably, high expression levels of *NRSN2-AS1* and *MCM3AP-AS1* have been shown to be associated with poor survival in liver cancer patients [17, 18]. In addition, high expression level of MIR429 has been shown to be correlated with larger tumor size and poor survival [19], while MIR210 was discovered to promote the malignant progression of liver cancer via autophagy, which might provide new directions for liver cancer therapy development [20].

Somatic mutation data helps to separate tumors in PartIES subtype 1 from other subtypes. Subtype 1 has the worst survival among all PartIES subtypes and has relatively high mutation burdens on important liver cancer genes (Figure S5f). For example, 50.0% of tumors in PartIES subtype 1 have mutations in gene *MUC16*, compared to a maximum of 13.8% in other PartIES subtypes. Mutations in *MUC16* have been shown to promote the metastasis of liver cancer cells[21]. Moreover, 22.2% of tumors in PartIES subtype 1 have LRP1B mutations compared to a maximum of 10.0% in other subtypes. LRP1B mutations are associated with the infiltration of immune cells in the microenvironment of liver cancer [22]. These observations are consistent with a recent review on liver cancer [23] which shows that a high tumor mutation burden is more likely to be associated with worse survival.

Investigate subtype-differentiated important genes using the PPI network

To investigate biological meanings of the 4 LIHC subtypes identified by PartIES, we examined omics features that differentiate them at a Bonferroni-adjusted P-value threshold 0.01 of KW tests. There are 13,458 differentially expressed mRNAs, 4,293 lncRNAs, 80 miRNAs, 97,254 differentially methylated CpG sites, and 292 differentially mutated genes. To identify important genes that interact with other genes based on the PPI network, we further selected the top 200 differentially expressed mRNAs (mapped to 199 genes in the PPI network), the top 200 lncRNAs (mapped to 3 genes in the PPI network), the top 80 differentially expressed miRNAs (no gene is mapped in

the PPI network), the top 200 differentially methylated CpG sites which correspond to 180 genes (mapped to 90 genes in the PPI network), and the top 200 differentially mutated genes (mapped all 200 genes in the PPI network). After removing overlapping genes, we ended up with 490 genes with 1,509 edges.

We similarly used degree, stress, and betweenness centrality to quantify interactions among these 490 genes. We only included genes in the largest sub-network (including 376 out of the 490 nodes) in comparison. Table S1 displays top 10 genes ranked by each of the 3 metrics. Genes ranked on top are potentially important LIHC genes. For example, gene *U2AF2*, which has the highest degree among all mapped genes, and gene *ABL1*, which has the second to highest stress and third to highest betweenness centrality among all mapped genes, have relatively high expression levels in tumors of PartIES subtype 4 (Figure S5b). High expression levels of these 2 genes are associated with poor liver cancer survival [24, 25].

Table S1: Top 10 genes ranked by degree, stress, and betweenness centrality for LIHC subtypes

Gene	Degree	Gene	Stress	Gene	Betweenness Centrality
U2AF2	49	GRB2	66590	ANK1	0.088
DHX15	46	ABL1	63498	GRB2	0.080
DHX9	45	ANK1	62894	ABL1	0.080
NCBP2	43	CSNK2A1	55624	CSNK2A1	0.074
HNRNPA2B1	43	CD44	55192	CD44	0.071
HNRNPK	41	CREB1	53660	CREB1	0.063
SF3B1	41	KHDRBS1	45188	RAB11A	0.060
SF1	39	HNRNPK	44674	GNGT2	0.056
EWSR1	38	RAB11A	42914	SMAD2	0.045
CPSF6	38	PSME3	41312	PARP1	0.0428

Pathway activities of cancer-related pathways of the 4 LIHC subtypes by PartIES

Figure S7 displays activities of six cancer-related pathways which have the most significantly different activities across the 4 LIHC subtypes. Note that activity scores of Hypoxia, p53, MAPK, TGFb, and NFkB pathways are relatively high in tumors in PartIES subtype 4, which has second to worst survival among all LIHC tumor samples. Studies show that these pathways play important roles in LIHC tumor genesis and development: alterations in p53 pathway activity and activation of the MAPK pathway both contribute to aggressiveness and progression in liver cancer [26, 27], and over-activation of TGFb pathway is highly likely to contribute to tumor progression in later stages in LIHC tumors [28]; Treatments targeting the transcription factor of Hypoxia have shown promising health outcomes in liver cancer patients [29], and NFkB pathway has been observed to play an important role in the relationship between inflammation and liver cancer development [30].

C.1.3 TCGA THCA

Using five omics data types, PartIES identified four THCA subtypes. Figure S8(a) presents Kaplan-Meier curves of the 4 THCA subtypes identified by PartIES. Subtype 3 containing 78 tumors has the worst survival with a 10-year survival rate of 0.78. Subtype 1 containing 97 tumors has the

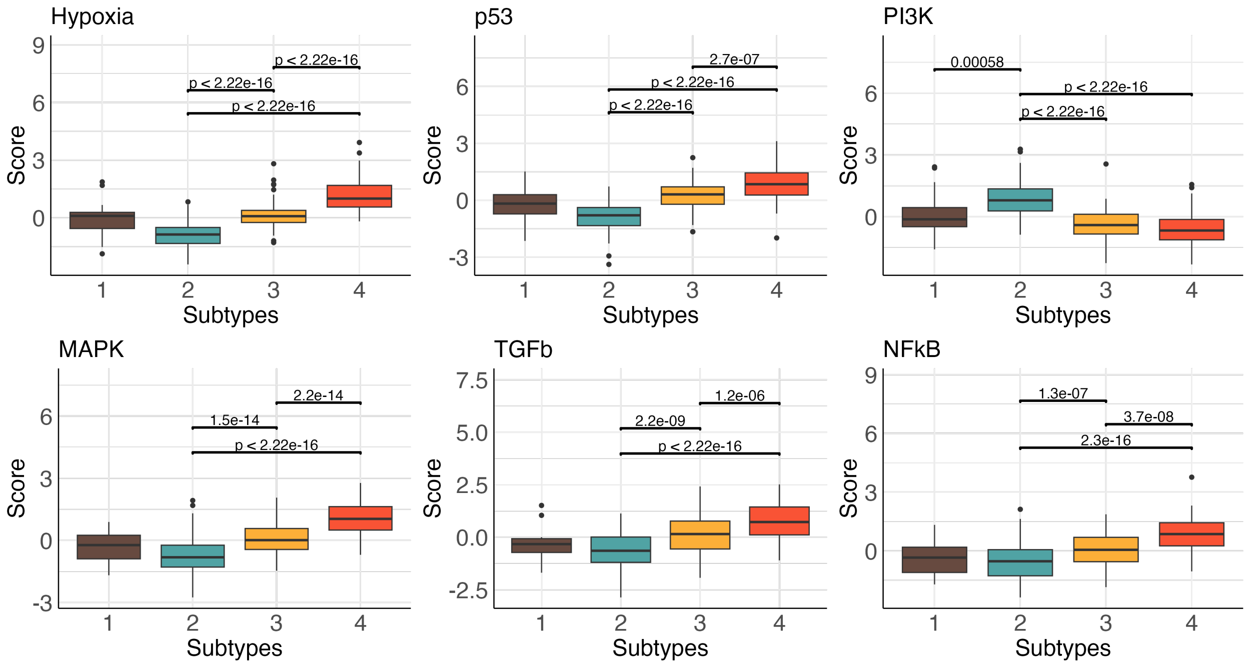

Figure S7: Boxplot of top 6 cancer-related pathways' activity scores ranked by KW test comparing scores across the four LIHC subtypes by PartIES. Also displayed are the P-values of the 3 pairs of subtypes with the most significant difference from the Wilcoxon rank sum test comparing pathway activities between each pair of subtypes.

best survival with a 10-year survival rate of 1. In the following sections, we first compared THCA subtypes identified by PartIES using five omics data types to those using one type of omics data. We then explored the biological meanings of the 4 PartIES THCA subtypes by finding important subtype-differentiated THCA genes on the PPI network and comparing cancer-related pathway activities across THCA subtypes.

TCGA THCA subtyping using a single omics data type vs. five omics data types

Figure S8(b)-S8(e) display the heatmaps of top 500 features in mRNA, lncRNA, miRNA expression levels, and DNA methylation levels ranked by P-values of Kruskal-Wallis (KW) test comparing features across the 4 THCA subtypes by PartIES. Figure S8(f) displays the heatmap of mutation profiles of the top 20 most frequently mutated genes across all 484 THCA tumors. Subtypes identified by individual omics data types using spectral clustering were also indicated. We note that 2 subtypes were identified using mRNA expression data only, 2 were identified using lncRNA expression data only, 2 were identified using miRNA expression data only, 4 were identified using DNA methylation data only, and 2 were identified using mutation data only. This information was further displayed in Figure S9.

Overall, DNA methylation data can separate subtype 1, from subtype 2, from subtypes 3 and 4 by PartIES, while lncRNA and miRNA expression data can further separate subtype 3 from subtype 4. Specific details of subtype comparisons and important THCA features differentiated the 4 PartIES THCA subtypes are provided below.

DNA methylation data separate tumor samples in PartIES subtype 1 and PartIES subtype 2 from other THCA samples. Specifically, 85.6% of tumors in PartIES subtype 1 are in the DNA methylation subtype 1, all tumors in PartIES subtype 2 are in the DNA methylation subtype 4. As shown in Figure S8, the top diffentiated CpG sites are highly methylated in THCA samples in PartIES subtype 2 and are near important THCA genes, such as cg05006942 near *NOB1* gene and cg13458212 near *PDLIM3* gene. These genes are associated with THCA tumor progression and metastasis [31, 32].

LncRNA and miRNA expression data help to further separate PartIES subtype 3 from subtype 4. Specifically, most tumors in PartIES subtype 4 are in the lncRNA subtype 1 (65.3%) and miRNA subtype 1(74.8%), while fewer tumors in PartIES subtype 3 belongs to lncRNA subtype 1(23.1%) and miRNA subtype 1(42.3%). Several lncRNAs with different expression levels across the 4 PartIES THCA subtypes, such as LINC01281 and LINC01586 (Figure S8c), were identified as differentially expressed lncRNAs in THCA tumor and normal samples in previous studies [33]. Similarly, for miRNAs, MIR7-2 and MIR146B have different expression levels across the 4 PartIES THCA subtypes (Figure S8d) and were also shown to be differentially expressed in THCA tumor and normal samples in previous studies[34, 35].

Investigate subtype-differentiated important genes using the PPI network

To investigate biological meanings of the 4 THCA subtypes identified by PartIES, we examined omics features that differentiate them at a Bonferroni-adjusted P-value threshold 0.01 of KW tests. There are 7,925 differentially expressed mRNAs, 3,426 lncRNAs, 37 miRNAs, 266,791 differentially methylated CpG sites, and 3 differentially mutated genes. To identify important genes that interact with other genes based on the PPI network, we further selected the top 200 differentially expressed mRNAs (mapped to 199 genes in the PPI network), the top 200 lncRNAs (mapped to 2 genes in the PPI network), all 37 differentially expressed miRNAs (mapped to 1 gene in the PPI network), the top 200 differentially methylated CpG sites which correspond to 194 genes (mapped to 185 genes in the PPI network), and all 3 differentially mutated genes (mapped to 3 genes in the PPI network). After removing overlapping genes, we ended up with 386 genes with 2,611 edges.

We similarly used degree, stress, and betweenness centrality to quantify interactions among these 386 mapped genes. We only included genes in the largest sub-network (including 289 out of the 386 nodes) in comparison. Table S2 displays top 10 genes ranked by each of the 3 metrics. Genes ranked on top are potentially important THCA genes. For example, gene *PTPRC* has highest degree, stress and betweenness centrality among all mapped genes and high expression levels of this gene is associated with poor prognosis of thyroid cancer [36]. Consistently, we can see that tumors in PartIES subtype 3, which has the worst survival among all THCA subtypes, have relatively high expression levels of gene *PTPRC* (Figure S8b).

Pathway activities of cancer-related pathways of the 4 THCA subtypes by PartIES

Figure S10 displays activities of six cancer-related pathways which have the most significantly different activities across the 4 THCA subtypes. Note that activity scores of all 6 displayed pathways are relatively low in tumors in PartIES subtype 3, which has the worst survival among all THCA tumor samples, and the activity scores of all 6 pathways are relatively high in tumors in PartIES subtype 2, which has the second to worst best survival among all THCA tumor samples. This

24222-4, partIES first cluster label

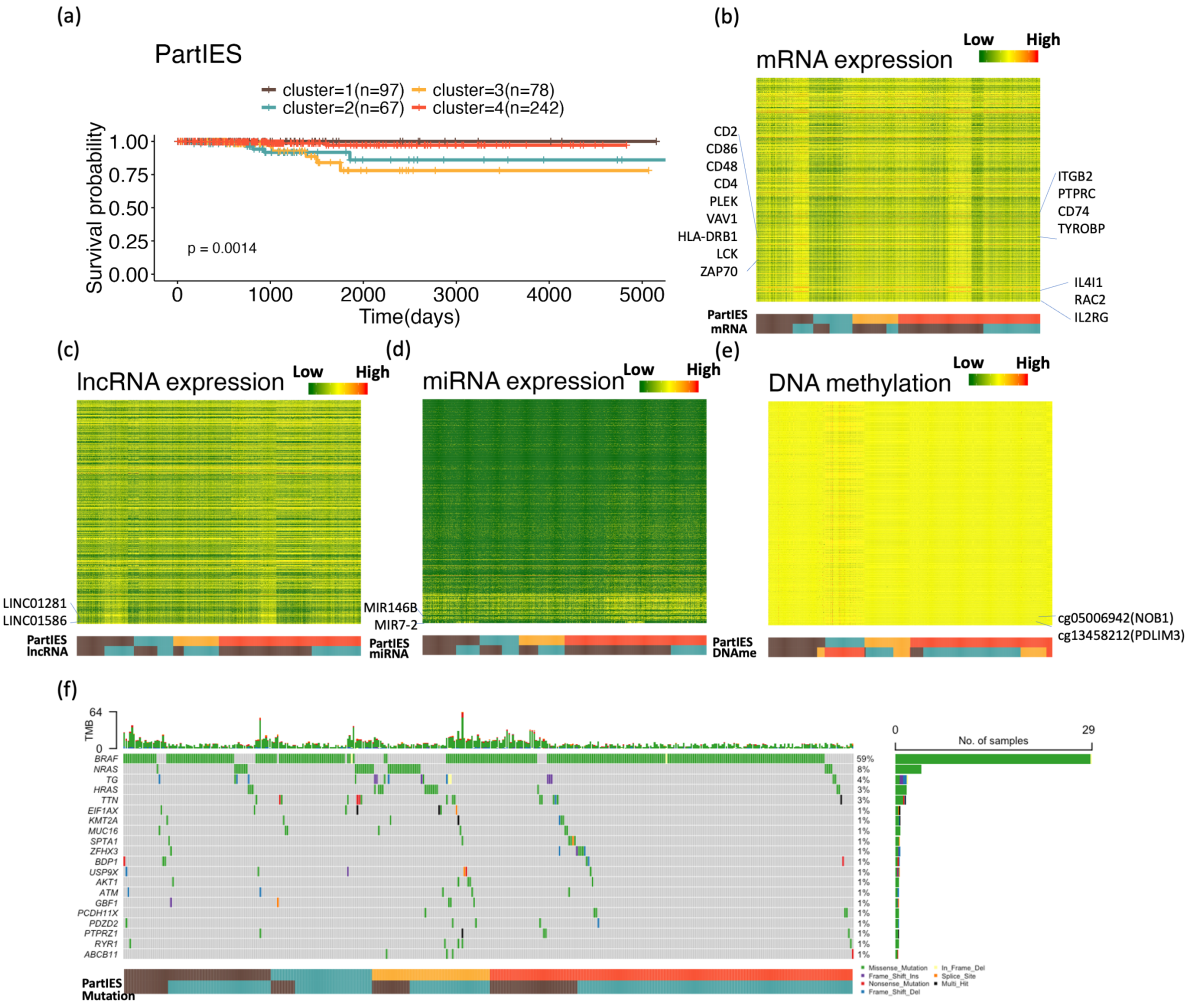

Figure S8: (a) Kaplan-Meier curves of the 4 TCGA THCA subtypes by PartIES. (b-e) Heatmaps of top 500 features by KW test comparing feature measures across the 4 THCA subtypes. (f) Mutation landscape of top 20 most frequently mutated genes across all THCA tumors.

indicates that these two subtypes, which have worse survival than other subtypes, might have different biological mechanisms in thyroid cancer development.

C.2 Sensitivity Analyses with fewer omics data types for LIHC and THCA

We conducted sensitivity analyses using subsets of the five omics data types for the 3 cancer types. Specifically, we examined PartIES and competing methods when 1) removing data types with similar clustering structures, and 2) removing data types with distinct clustering structures. Details of the analyses and results for BLCA has been summarized in Table 4 in the main text.

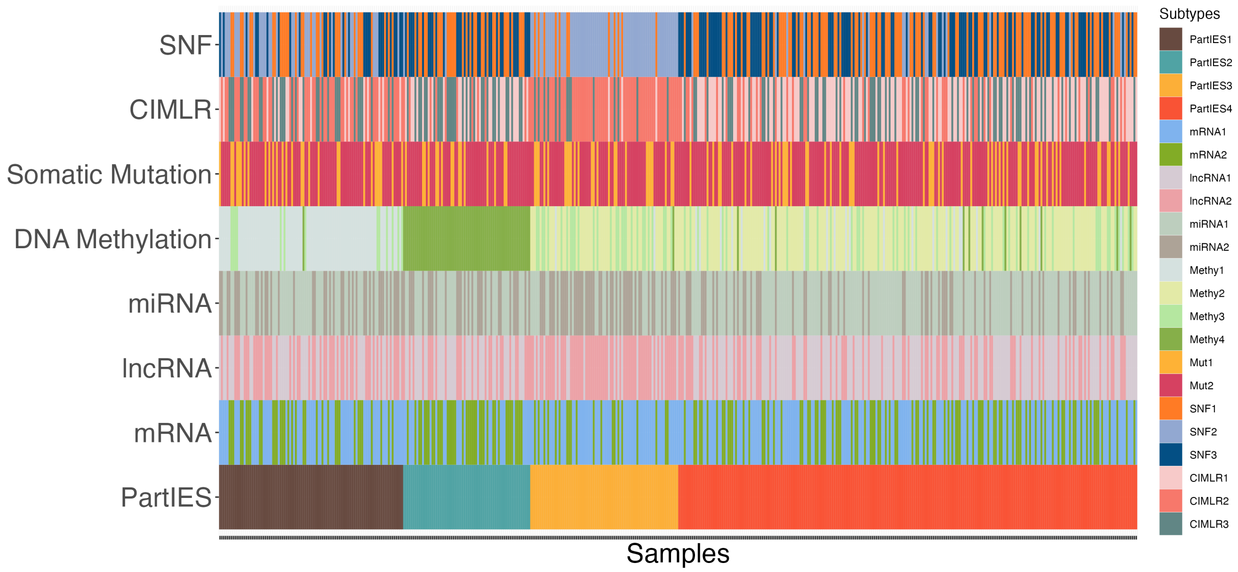

Figure S9: TCGA THCA subtypes identified by PartIES and competing methods using five types of omics data vs. that using one type of omics data. Samples are ordered by PartIES subtypes.

Table S2: Top 10 genes ranked by degree, stress, and betweenness centrality for THCA subtypes

Gene	Degree	Gene	Stress	Gene	Betweenness Centrality
PTPRC	115	PTPRC	73716	PTPRC	0.10556638
CD4	99	CD4	58062	PLEK	0.0819596
ITGB2	87	LCK	54416	CD4	0.08002822
LCK	79	PLEK	48956	LCK	0.06500318
CD86	78	ITGB2	44358	ITGB2	0.04588748
CD48	70	VAV1	34062	BTK	0.04030841
CD2	69	BTK	33416	SPI1	0.03943823
BTK	67	SPI1	32908	RUNX3	0.03898052
CTLA4	66	RAC2	32380	RAC2	0.03779241
VAV1	66	CD74	30994	WAS	0.037595

Here we display the results for LIHC and THCA

For LIHC, from Figure S6, we can see that mRNA expression and somatic mutation data provide distinct clustering structures, while lncRNA expression data, miRNA expression data and DNA methylation data provide similar cluster information. Therefore, we repeated the analyses removing i) miRNA data, ii) miRNA and DNA methylation data, iii) mRNA data, and iv) mRNA and mutation data.

For THCA, from Figure S9, we can see that DNA methylation data provides distinct clustering structures, lncRNA and miRNA provide similar cluster structure, and mRNA and mutation data provide similar cluster structures. Therefore, we repeated the analyses removing i) mutation data, ii) mutation and mRNA data, iii) lncRNA data, and iv) lncRNA and DNA methylation data.

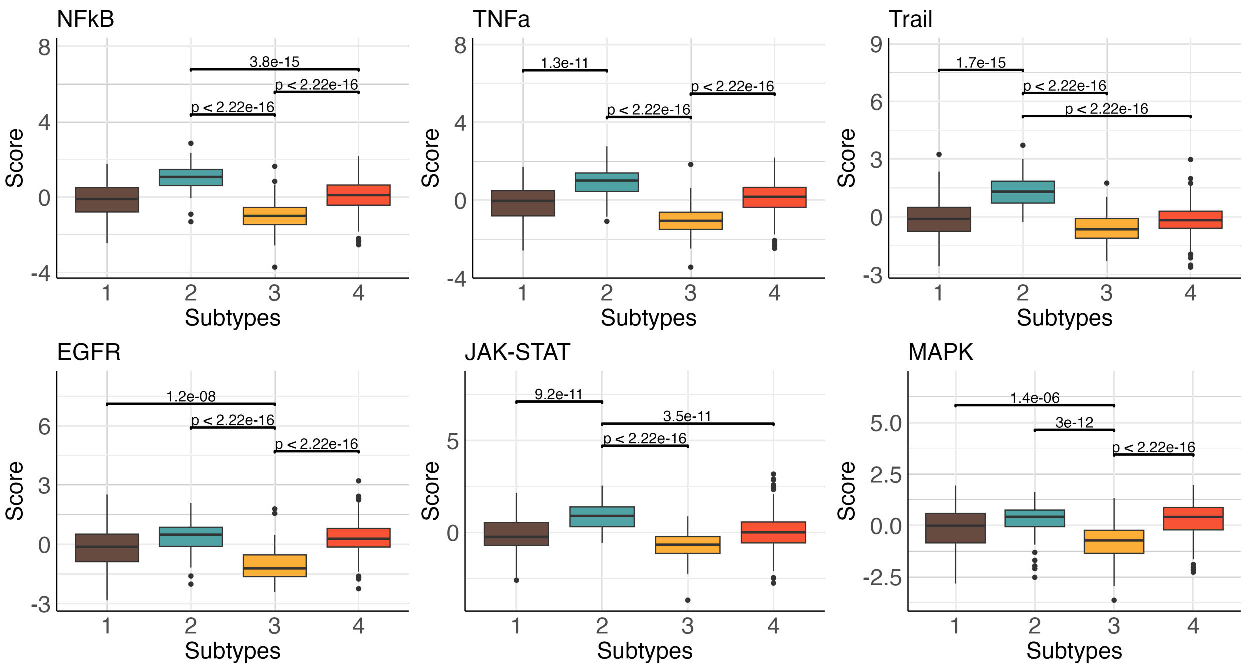

Figure S10: Boxplot of top 6 cancer-related pathways' activity scores ranked by KW test comparing scores across the four THCA subtypes by PartIES. Also displayed are the P-values of the 3 pairs of subtypes with the most significant difference from the Wilcoxon rank sum test comparing pathway activities between each pair of subtypes.

Results of sensitivity analyses using subsets of 5 types of omics data for LIHC and THCA are summarized in table S3 and S4 separately. Similar results have been observed from the sensitivity analyses for LIHC and THCA as those for BLCA in the main text.

Table S3: Sensitivity analysis of TCGA LIHC subtyping with different omics data types

**Cancer**	**Analysis**	**Data types**		**PartIES**	**CIMLR**	**SNF**
LIHC	1	mRNA, mutation, LncRNA, DNA methylation, miRNA	Number of clusters	4	3	2
			Survival P-value	9.08E-04	0.1915	7.81E-03
	2	mRNA, mutation, LncRNA, DNA methylation	Number of clusters	4	3	2
			Survival P-value	9.86E-04	0.1891	0.0214
	3	mRNA, mutation, LncRNA	Number of clusters	4	3	2
			Survival P-value	2.65E-04	0.3772	0.1075
	4	mutation, LncRNA, DNA methylation, miRNA	Number of clusters	3	3	2
			Survival P-value	0.2118	0.2625	4.06E-03
	5	LncRNA, DNA methylation, miRNA	Number of clusters	3	3	2
			Survival P-value	0.2548	0.2745	3.84E-03

D Computational time

We examined the computational time of PartIES and competing methods. With a machine of Apple M1 Pro 8 core CPU @ 3.2 GHz, the computation times in seconds of PartIES, CIMLR, and SNF for the three TCGA applications are shown in Table S5.

Table S4: Sensitivity analysis of TCGA THCA subtyping with different omics data types

**Cancer**	**Analysis**	**Data types**		**PartIES**	**CIMLR**	**SNF**
THCA	1	DNA methylation, miRNA, lncRNA, mRNA, mutation	Number of clusters	4	3	3
			Survival P-value	1.39E-03	0.6404	0.9297
	2	DNA methylation, miRNA, lncRNA, mRNA	Number of clusters	4	3	3
			Survival P-value	5.08E-03	0.8403	0.9035
	3	DNA methylation, miRNA, lncRNA	Number of clusters	4	3	3
			Survival P-value	5.03E-03	0.3362	0.0265
	4	DNA methylation, miRNA, mRNA, mutation	Number of clusters	4	3	3
			Survival P-value	5.03E-03	0.2883	0.6655
	5	miRNA, mRNA, mutation	Number of clusters	3	3	3
			Survival P-value	0.0114	0.3581	0.3981

Table S5: Computational time of PartIES and competing methods in three TCGA applications

Cancer	N	Diffusion time	SNF time	CIMLR time	PartIES time
BLCA	401	0.18	10.23	13.68	39.75
LIHC	362	0.21	7.56	10.13	32.02
THCA	484	0.20	14.64	19.29	59.97

References

[1] Stephen P. Boyd and Lieven Vandenberghe. *Convex Optimization*. Cambridge University Press, Cambridge, UK ; New York, 2004.

[2] Feiping Nie, Xiaoqian Wang, Michael Jordan, and Heng Huang. The Constrained Laplacian Rank Algorithm for Graph-Based Clustering. *Proceedings of the AAAI Conference on Artificial Intelligence*, 30(1), March 2016.

[3] Daniele Ramazzotti, Avantika Lal, Bo Wang, Serafim Batzoglou, and Arend Sidow. Multi-omic tumor data reveal diversity of molecular mechanisms that correlate with survival. *Nature Communications*, 9(1):4453, October 2018.

[4] Abhi Veerakumarasivam, Helen E. Scott, Suet-Feung Chin, Anne Warren, Matthew J. Wallard, Donna Grimmer, Koichi Ichimura, Carlos Caldas, V. Peter Collins, David E. Neal, and John D. Kelly. High-Resolution Array-Based Comparative Genomic Hybridization of Bladder Cancers Identifies Mouse Double Minute 4 (MDM4) as an Amplification Target Exclusive of MDM2 and TP53. *Clinical Cancer Research*, 14(9):2527–2534, May 2008.

[5] Yang Peng, Wen Dong, Tian-xin Lin, Guang-zheng Zhong, Bei Liao, Bo Wang, Peng Gu, Li Huang, Yun Xie, Fu-ding Lu, Xu Chen, Wei-bin Xie, Wang He, Shao-xu Wu, and Jian Huang. MicroRNA-155 promotes bladder cancer growth by repressing the tumor suppressor DMTF1. *Oncotarget*, 6(18):16043–16058, April 2015.

[6] Zhuolun Sun, Changying Jing, Hailun Zhan, Xudong Guo, Ning Suo, Feng Kong, Wen Tao, Chutian Xiao, Daoyuan Hu, Hanbo Wang, and Shaobo Jiang. Identification of tumor antigens and immune landscapes for bladder urothelial carcinoma mRNA vaccine. *Frontiers in Immunology*, 14:1097472, January 2023.

[7] Weisheng Li, Yang Xiong, Junlei Zhu, Xiaoxiao Jin, Jin Meng, and Wenqiang He. Establishing a prognostic model with ferroptosis-related long non-coding RNAs in bladder cancer. *Translational Cancer Research*, 12(8), August 2023.

[8] Zuwei Li, Yuwu Li, Weizhang Zhong, and Peiyuan Huang. m6A-Related lncRNA to Develop Prognostic Signature and Predict the Immune Landscape in Bladder Cancer. *Journal of Oncology*, 2021:7488188, July 2021.

[9] Chia-Lun Wu, Jar-Yi Ho, Shun-Hsing Hung, and Dah-Shyong Yu. miR-429 expression in bladder cancer and its correlation with tumor behavior and clinical outcome. *The Kaohsiung Journal of Medical Sciences*, 34(6):335–340, June 2018.

[10] Jun Feng Liang, Pei Hua Li, Yong Zhu, Shuai Shuai Zheng, Jing Wei Liu, and Shi Qiang Song. MicroRNA-186 suppresses cell proliferation and metastasis in bladder cancer. *African Health Sciences*, 22(4):56–63, December 2022.

[11] WenBo Wu, GaoZhen Jia, Lei Chen, HaiTao Liu, and ShuJie Xia. Analysis of the Expression and Prognostic Value of Annexin Family Proteins in Bladder Cancer. *Frontiers in Genetics*, 12:731625, August 2021.

[12] Jian-Yun Xie, Peng-Chen Chen, Jia-Li Zhang, Ze-Shou Gao, Henrique Neves, Shu-Dong Zhang, Qing Wen, Wei-Dong Chen, Hang Fai Kwok, and Yao Lin. The prognostic significance of DAPK1 in bladder cancer. *PLoS ONE*, 12(4):e0175290, April 2017.

[13] A. Gordon Robertson, Jaegil Kim, Hikmat Al-Ahmadie, Joaquim Bellmunt, Guangwu Guo, Andrew D. Cherniack, Toshinori Hinoue, Peter W. Laird, Katherine A. Hoadley, Rehan Akbani, Mauro A.A. Castro, Ewan A. Gibb, Rupa S. Kanchi, Dmitry A. Gordenin, Sachet A. Shukla, Francisco Sanchez-Vega, Donna E. Hansel, Bogdan A. Czerniak, Victor E. Reuter, Xiaoping Su, Benilton De Sa Carvalho, Vinicius S. Chagas, Karen L. Mungall, Sara Sadeghi, Chandra Sekhar Pedamallu, Yiling Lu, Leszek J. Klimczak, Jiexin Zhang, Caleb Choo, Akinyemi I. Ojesina, Susan Bullman, Kristen M. Leraas, Tara M. Lichtenberg, Catherine J. Wu, Nicholaus Schultz, Gad Getz, Matthew Meyerson, Gordon B. Mills, David J. McConkey, John N. Weinstein, David J. Kwiatkowski, Seth P. Lerner, Rehan Akbani, Hikmat Al-Ahmadie, Monique Albert, Iakovina Alexopoulou, Adrian Ally, Tatjana Antic, Manju Aron, Miruna Balasundaram, John Bartlett, Stephen B. Baylin, Allison Beaver, Joaquim Bellmunt, Inanc Birol, Lori Boice, Moiz S. Bootwalla, Jay Bowen, Reanne Bowlby, Denise Brooks, Bradley M. Broom, Wiam Bshara, Susan Bullman, Eric Burks, Flavio M. Cárcano, Rebecca Carlsen, Benilton S. Carvalho, Andre L. Carvalho, Eric P. Castle, Mauro A.A. Castro, Patricia Castro, James W. Catto, Vinicius S. Chagas, Andrew D. Cherniack, David W. Chesla, Caleb Choo, Eric Chuah, Sudha Chudamani, Victoria K. Cortessis, Sandra L. Cottingham, Daniel Crain, Erin Curley, Bogdan A. Czerniak, Siamak Daneshmand, John A. Demchok, Noreen Dhalla, Hooman Djaladat, John Eckman, Sophie C. Egea, Jay Engel, Ina Felau, Martin L. Ferguson, Johanna Gardner, Julie M. Gastier-Foster, Mark Gerken, Gad Getz, Ewan A. Gibb, Carmen R. Gomez-Fernandez, Dmitry A. Gordenin, Guangwu Guo, Donna E. Hansel, Jodi Harr, Arndt Hartmann, Lynn M. Herbert, Toshinori Hinoue, Thai H. Ho, Katherine A. Hoadley, Robert A. Holt, Carolyn M. Hutter, Steven J.M. Jones, Merce Jorda, Richard J. Kahnoski, Rupa S. Kanchi, Katayoon Kasaian, Jaegil Kim, Leszek J. Klimczak, David J. Kwiatkowski, Phillip H. Lai, Peter W. Laird, Brian R. Lane, Kristen M. Leraas, Seth P. Lerner, Tara M. Lichtenberg, Jia Liu, Laxmi Lolla, Yair Lotan, Yiling Lu, Fabiano R. Lucchesi, Yussanne Ma, Roberto D. Machado, Dennis T. Maglinte, David Mallery, Marco A. Marra, Sue E. Martin, Michael Mayo, David J. McConkey, Anoop Meraney, Matthew Meyerson, Gordon B. Mills, Alireza Moinzadeh, Richard A. Moore, Edna M. Mora Pinero, Scott Morris, Carl Morrison, Karen L. Mungall, Andrew J. Mungall, Jerome B. Myers, Rashi Naresh, Peter H. O'Donnell, Akinyemi I. Ojesina, Dipen J. Parekh, Jeremy Parfitt, Joseph D. Paulauskis,

Chandra Sekhar Pedamallu, Robert J. Penny, Todd Pihl, Sima Porten, Mario E. Quintero-Aguilo, Nilsa C. Ramirez, W. Kimryn Rathmell, Victor E. Reuter, Kimberly Rieger-Christ, A. Gordon Robertson, Sara Sadeghi, Charles Saller, Andrew Salner, Francisco Sanchez-Vega, George Sandusky, Cristovam Scapulatempo-Neto, Jacqueline E. Schein, Anne K. Schuckman, Nikolaus Schultz, Candace Shelton, Troy Shelton, Sachet A. Shukla, Jeff Simko, Parminder Singh, Payal Sipahimalani, Norm D. Smith, Heidi J. Sofia, Andrea Sorcini, Melissa L. Stanton, Gary D. Steinberg, Robert Stoehr, Xiaoping Su, Travis Sullivan, Qiang Sun, Angela Tam, Roy Tarnuzzer, Katherine Tarvin, Helge Taubert, Nina Thiessen, Leigh Thorne, Kane Tse, Kelinda Tucker, David J. Van Den Berg, Kim E. Van Kessel, Sven Wach, Yunhu Wan, Zhining Wang, John N. Weinstein, Daniel J. Weisenberger, Lisa Wise, Tina Wong, Ye Wu, Catherine J. Wu, Liming Yang, Leigh Anne Zach, Jean C. Zenklusen, Jiashan (Julia) Zhang, Jiexin Zhang, Erik Zmuda, and Ellen C. Zwarthoff. Comprehensive Molecular Characterization of Muscle-Invasive Bladder Cancer. *Cell*, 171(3):540–556.e25, October 2017.

[14] Yanqing Li, Yan Jiao, Yang Li, and Yanan Liu. Expression of La Ribonucleoprotein Domain Family Member 4B (LARP4B) in Liver Cancer and Their Clinical and Prognostic Significance. *Disease Markers*, 2019:1569049, October 2019.

[15] Sheng Tan, Ming Zhang, Xinglong Shi, Keshuo Ding, Qiang Zhao, Qianying Guo, Hao Wang, Zhengsheng Wu, Yani Kang, Tao Zhu, Jielin Sun, and Xiaodong Zhao. CPSF6 links alternative polyadenylation to metabolism adaption in hepatocellular carcinoma progression. *Journal of Experimental & Clinical Cancer Research*, 40(1):85, March 2021.

[16] Xu Wang, Tao Zhou, Xingyi Chen, Yu Wang, Yushi Ding, Haoyang Tu, Shengyang Gao, Haoyu Wang, Xinying Tang, and Yong Yang. System analysis based on the cancer-immunity cycle identifies ZNF207 as a novel immunotherapy target for hepatocellular carcinoma. *Journal for Immunotherapy of Cancer*, 10(3):e004414, March 2022.

[17] Xing Feng Huang, Li Sheng Fu, Qian Qian Cai, and Fei Fan. Prognostic and immunological role of sulfatide-related lncRNAs in hepatocellular carcinoma. *Frontiers in Oncology*, 13:1091132, February 2023.

[18] Tao Ma, Fa-Hong Wu, Hong-Xia Wu, Qiong Fa, and Yan Chen. Long Non-Coding RNA MCM3AP-AS1: A Crucial Role in Human Malignancies. *Pathology and Oncology Research*, 28:1610194, June 2022.

[19] Xiao-Ying Huang, Jin-Guang Yao, Hong-Dong Huang, Chao Wang, Yun Ma, Qiang Xia, and Xi-Dai Long. MicroRNA-429 Modulates Hepatocellular Carcinoma Prognosis and Tumorigenesis. *Gastroenterology Research and Practice*, 2013:804128, 2013.

[20] Shumin Bi, Yidan Zhang, Jia Zhou, Yuanyuan Yao, Jiadong Wang, Miaomiao Fang, Baozhu Li, Changhao Wu, and Chunxia Ren. miR-210 promotes hepatocellular carcinoma progression by modulating macrophage autophagy through PI3K/AKT/mTOR signaling. *Biochemical and Biophysical Research Communications*, 662:47–57, June 2023.

[21] Yao Huang, Xiaoyu Huang, Jianxing Zeng, and Jun Lin. Knockdown of MUC16 (CA125) Enhances the Migration and Invasion of Hepatocellular Carcinoma Cells. *Frontiers in Oncology*, 11, 2021.

[22] Xiangyu Zhai, Zhijia Xia, Gang Du, Xinlu Zhang, Tong Xia, Delin Ma, Xiaosong Li, Bin Jin, and Hao Zhang. LRP1B suppresses HCC progression through the NCSTN/PI3K/AKT sig-

naling axis and affects doxorubicin resistance. *Genes & Diseases*, 10(5):2082–2096, September 2023.

[23] Daniela Gabbia and Sara De Martin. Tumor Mutational Burden for Predicting Prognosis and Therapy Outcome of Hepatocellular Carcinoma. *International Journal of Molecular Sciences*, 24(4):3441, February 2023.

[24] Jing Cao, Cuicui Xiao, Christ-Jonathan Tsia Hin Fong, Jiao Gong, Danyang Li, Xiangyong Li, Yusheng Jie, and Yutian Chong. Expression and Regulatory Network Analysis of Function of Small Nucleolar RNA Host Gene 4 in Hepatocellular Carcinoma. *Journal of Clinical and Translational Hepatology*, 10(2):297–307, April 2022.

[25] Fang Wang, Wei Hou, Lennox Chitsike, Yingchen Xu, Carlee Bettler, Aldeb Perera, Thomas Bank, Scott J. Cotler, Asha Dhanarajan, Mitchell F. Denning, Xianzhong Ding, Peter Breslin, Wenan Qiang, Jun Li, Anthony J. Koleske, and Wei Qiu. ABL1, Overexpressed in Hepatocellular Carcinomas, Regulates Expression of NOTCH1 and Promotes Development of Liver Tumors in Mice. *Gastroenterology*, 159(1):289–305.e16, July 2020.

[26] Xuan Meng, Derek A. Franklin, Jiahong Dong, and Yanping Zhang. MDM2–p53 Pathway in Hepatocellular Carcinoma. *Cancer Research*, 74(24):7161–7167, December 2014.

[27] Bénédicte Delire and Peter Stärkel. The Ras/MAPK pathway and hepatocarcinoma: Pathogenesis and therapeutic implications. *European Journal of Clinical Investigation*, 45(6):609–623, June 2015.

[28] Isabel Fabregat, Joaquim Moreno-Càceres, Aránzazu Sánchez, Steven Dooley, Bedair Dewidar, Gianluigi Giannelli, Peter ten Dijke, and the IT-LIVER Consortium. TGF-β signalling and liver disease. *The FEBS Journal*, 283(12):2219–2232, 2016.

[29] Cressida Bowyer, Andrew L. Lewis, Andrew W. Lloyd, Gary J. Phillips, and Wendy M. Macfarlane. Hypoxia as a target for drug combination therapy of liver cancer. *Anti-Cancer Drugs*, 28(7):771–780, August 2017.

[30] Pablo Muriel. NF-κB in liver diseases: A target for drug therapy. *Journal of Applied Toxicology*, 29(2):91–100, 2009.

[31] Shan Lin, Wei Meng, Wei Zhang, Jia Liu, Peisong Wang, Shuai Xue, and Guang Chen. Expression of the NOB1 gene and its clinical significance in papillary thyroid carcinoma. *The Journal of International Medical Research*, 41(3):568–572, June 2013.

[32] Yue Huang, Yaoxin Wang, Sining Liu, Zhengmin Xu, and Wen-Xia Chen. An integrative analysis of the tumor suppressors and oncogenes from sexual dimorphism and gene expression alteration features in thyroid cancer. *Cancer Biomarkers: Section A of Disease Markers*, 38(1):1–16, 2023.

[33] Yun Zhang, Taobo Jin, Haipeng Shen, Junfeng Yan, Ming Guan, and Xin Jin. Identification of Long Non-Coding RNA Expression Profiles and Co-Expression Genes in Thyroid Carcinoma Based on The Cancer Genome Atlas (TCGA) Database. *Medical Science Monitor : International Medical Journal of Experimental and Clinical Research*, 25:9752–9769, December 2019.

[34] Hua-yu Wu, Yi Wei, and Shang-ling Pan. Down-regulation and clinical significance of miR-7-2-3p in papillary thyroid carcinoma with multiple detecting methods. *IET Systems Biology*, 13(5):225–233, August 2019.

[35] Daniel Casartelli de Santa-Inez, Cesar Seigi Fuziwara, Kelly Cristina Saito, and Edna Teruko Kimura. Targeting the Highly Expressed microRNA miR-146b with CRISPR/Cas9n Gene Editing System in Thyroid Cancer. *International Journal of Molecular Sciences*, 22(15):7992, July 2021.

[36] Yanmeizhi Wu, Jun Han, Kazakova Elena Vladimirovna, Shumei Zhang, Wenhua Lv, Yan Zhang, Esma Jamaspishvili, Jingxue Sun, Qingxiao Fang, Jingjing Meng, and Hong Qiao. Upregulation Of Protein Tyrosine Phosphatase Receptor Type C Associates To The Combination Of Hashimoto's Thyroiditis And Papillary Thyroid Carcinoma And Is Predictive Of A Poor Prognosis. *OncoTargets and therapy*, 12:8479–8489, October 2019.
